# Acute liver failure-induced arginine deficiency impairs blood-brain barrier via inhibiting mTORC1-S6K1/4EBP1 pathway and inducing autophagy

**DOI:** 10.1038/s41419-025-08152-4

**Published:** 2025-11-17

**Authors:** Hanyu Yang, Weimin Kong, Ling Jiang, Liang Zhu, Xun Wang, Lu Yang, Liqiang Qian, Zijun Xu, Chenyang Zhang, Xiaodong Liu, Li Liu

**Affiliations:** 1 Department of Pharmacology, School of Pharmacy, China Pharmaceutical University, Nanjing, China; 2 School of Pharmacy, Bengbu Medical University, Bengbu, China

**Keywords:** TOR signalling, Blood-brain barrier, Hepatitis

## Abstract

Blood-brain barrier (BBB) impairment plays a crucial role in the development of hepatic encephalopathy. Our previous work demonstrated that hepatic ischemia-reperfusion-induced acute liver failure (ALF) impairs the BBB by releasing arginase, but the underlying mechanism remains unclear. In this study, we discovered that ALF-induced arginase accumulation leads to arginine (Arg) deficiency, causing BBB cells to arrest in G1 phase. This arrest was associated with decreased expression of key cell cycle regulatory proteins, activation of autophagy, and inhibition of the mechanistic target of rapamycin complex 1 (mTORC1) pathway. Silencing mTORC1 downstream protein p70 ribosomal protein S6 kinase 1 (S6K1) or eukaryotic translation initiation factor 4E binding protein 1 (4EBP1) showed similar effects as Arg deficiency, while activating the mTORC1 pathway attenuated arginase-induced cell cycle delay. Furthermore, inhibition of autophagy with 3-methyladenine or silencing Beclin-1 partially reversed the arginase-induced effects. These in vitro findings were corroborated in rat models of ALF induced by thioacetamide or acetaminophen, as well as in rats treated with arginase, all of which exhibited elevated plasma arginase activity, reduced Arg levels, increased BBB permeability, and suppressed BBB cell proliferation. These changes were accompanied by alterations in markers related to cell cycles, mTORC1 signaling, and autophagy, which were reversible upon Arg supplementation. In summary, our research reveals that ALF-induced BBB damage is driven by Arg deprivation due to arginase release, leading to G1 phase arrest through mTORC1 pathway inhibition and autophagy induction, which provides new insights into the prevention and treatment of ALF-induced BBB damage and hepatic encephalopathy.

## Introduction

Acute liver failure (ALF) often leads to hepatic encephalopathy [1, 2], resulting from the accumulation of neurotoxins in the brain due to blood-brain barrier (BBB) dysfunction [1, 3]. Several studies have demonstrated that ALF impairs the BBB [4–6], but the underlying mechanisms are not fully understood. As ALF is often associated with substantial hepatic necrosis [7], a hypothesis was raised that some molecules are released from the injured liver, damaging the BBB. This hypothesis is supported by our previous studies showing that serum from ALF rats impairs rat or human brain microvascular endothelial cells [5, 8], the principal component of the BBB. After purification, we have identified this damage-associated molecule as arginase [8].

Arginases are abundant in the livers of mammals and can be released from hepatocytes into circulation, which efficiently catalyze the conversion of arginine (Arg) to ornithine and urea. Some endogenous substances, such as polyamines, proline, and nitric oxide (NO), are also substrates for arginases [9]. The relative stability of arginase content and activity is crucial for brain function. Arginase knockout mice exhibit worsened neurological deficits and increased brain infarction after stroke [10]. By contrast, Alzheimer’s disease-induced hippocampal neuronal death and memory loss are associated with extracellular arginase accumulation [11]. Significantly increased arginase activity has been detected in the serum of patients with liver injury [12–14] and animals with ALF [8, 15], accompanied by Arg deficiency [13, 16, 17]. Our previous research further revealed that ALF induced by hepatic ischemia-reperfusion impairs the BBB by releasing arginase from the injured liver [8]. However, the mechanisms by which ALF-mediated arginase release impairs BBB function remain unclear.

Excessive release of arginase can directly lead to Arg deficiency, and Arg deficiency participates in various pathophysiological processes, such as endothelial and immune cell dysfunction, as well as tumor cell death [18, 19]. Liver-specific deletion of *Atg7* produced circulating arginase and reduced both serum Arg levels and tumor growth. Dietary supplementation of *Atg7*-deficient hosts with Arg partially restored levels of circulating Arg and tumor growth [19]. Arg plays physiological roles mainly via the NO pathway or mechanistic target of rapamycin complex 1 (mTORC1) signaling [20]. NO, produced from Arg by NO synthetase, serves as a signaling molecule in the corresponding pathophysiological processes [21]. Dysfunction of the mTORC1 pathway is involved in some brain-related pathological events, such as traumatic brain injury [22] and cerebral stroke [23]. Arg activates the mTORC1 pathway by promoting mTORC1 translocation to the lysosome surface and regulating the phosphorylation of p70 ribosomal protein S6 kinase 1 (S6K1) at Thr389 (p-S6K1) and eukaryotic translation initiation factor 4E binding protein 1 (4EBP1) at Thr37/46 (p-4EBP1) [24]. Besides, mTORC1 inhibition also induces autophagy [25], and autophagy induction is involved in BBB impairment by cerebral ischemia [26] and the HIV protein Tat [27]. Generally, autophagy plays a protective role in cells, but disruption of autophagy mechanisms or excessive autophagic flux usually leads to cellular dysfunction and death [28]. Microtubule-associated protein 1 light chain 3 (LC3) is widely used to monitor autophagy [29]. During autophagy, cytosolic LC3 (LC3-I) is lipidated by phosphatidylethanolamine conjugation to form LC3-II, which embeds into the phagophore membrane during its expansion. Sequestosome 1 (p62) interacts with the LC3-II on the phagophore membrane, and this interaction facilitates the engulfment of these substrates by the expanding phagophore, which matures into a sealed autophagosome. Following autophagosome-lysosome fusion, both the cargo and p62 are degraded in the autolysosome [30, 31]. Thus, the autophagy process itself is also one of the clearance mechanisms for p62. Nutritional stress, such as amino acid deprivation or energy deficiency, will trigger autophagy activation through the mTORC1 [32] or the AMP-activated protein kinase (AMPK) signaling pathway, respectively [33]. L-glutamine uptake is regulated by SLC1A5, and loss of SLC1A5 function inhibits cell growth and activates autophagy via antagonizing the mTORC1 pathway [34]. We previously reported that Arg deprivation blocked BBB cell proliferation by arresting the cells in the G0/G1 phase [8]. The present study will further investigate the mechanism that Arg deficiency inhibits BBB cell proliferation and whether the impairment of BBB cells by Arg deficiency is involved in the mTORC1 or autophagy pathway.

The present study aimed to (i) investigate whether ALF impairs BBB by depriving Arg due to substantial arginase release using different ALF rat models and primary cultured rat brain microvascular endothelial cells (rBMECs); (ii) investigate whether Arg deficiency impairs BBB via the mTORC1 or autophagy pathway using hCMEC/D3 cells as an in vitro BBB model. The thioacetamide (TAA)-induced ALF rats, acetaminophen (APAP)-induced ALF rats, and arginase-treated rats were used to confirm the in vitro findings. These results will emphasize the importance of ALF-induced Arg deficiency in BBB dysfunction and provide new insights into the prevention and treatment of ALF-induced BBB damage.

## Results

### ALF-induced Arg deficiency impaired brain microvascular endothelial cells via retarding cells in G1 phase

**Fig. 1:**
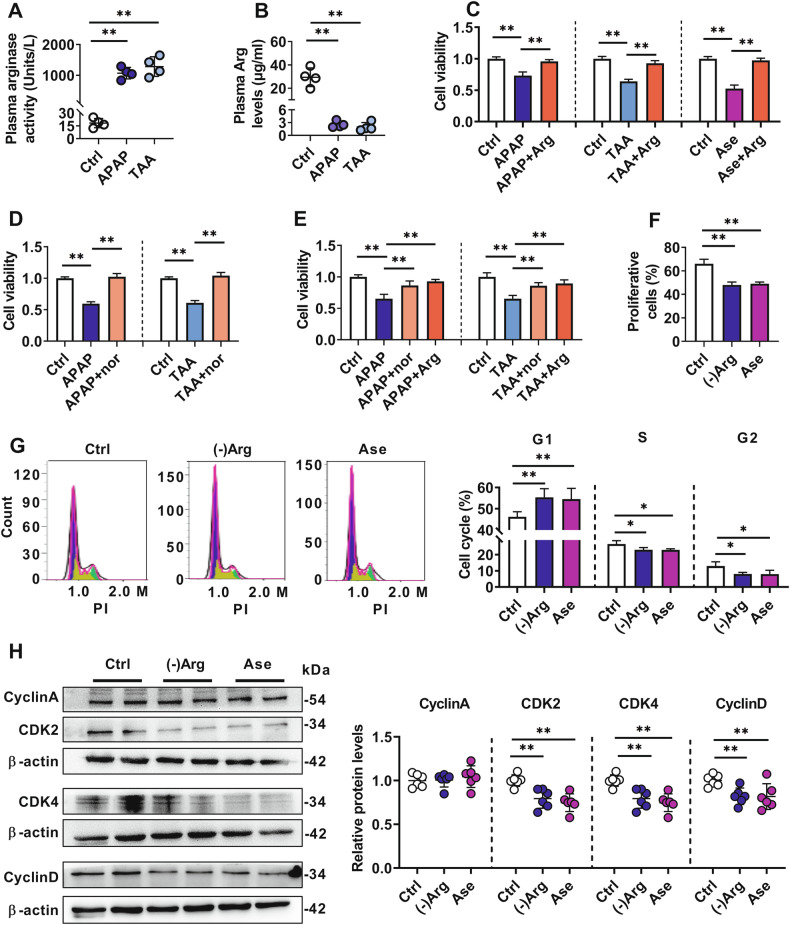
Damage effects of arginase in serum of acute liver failure (ALF) rats on hCMEC/D3 cells and rBMECs. Arginase (Ase) activity (**A**) and arginine (Arg) levels (**B**) in plasma of acetaminophen (APAP)- or thioacetamide (TAA)-induced ALF rats (*n* = 4). **C** Effects of 50 μg/mL Arg supplementation on hCMEC/D3 cell damage caused by culture medium containing 10% APAP/TAA rat serum or 20 μg/mL Ase (*n* = 6). **D** Effects of Ase inhibitor nor-NOHA (nor) (50 μM) on hCMEC/D3 cell damage caused by culture medium containing 10% APAP or TAA rat serum (*n* = 6). **E** Effects of nor-NOHA or Arg on rBMECs damage caused by culture medium containing 50% APAP or TAA rat serum (*n* = 6). Cell proliferation assay by EdU incorporation (**F**) and cell cycle analysis on flow cytometer by PI staining (**G**) of rBMECs cells treated with arginine-free culture ((-)Arg) or 20 μg/mL Ase (*n* = 4). **H** Western blot of cell cycle-related proteins in rBMECs treated with (-)Arg or Ase (*n* = 6). Data are expressed as mean ± SD. *p < 0.05; **p < 0.01. Statistical significance was determined with the 1-way ANOVA followed by the Dunnett post hoc test.

Toxic effects of both serums of the APAP- and TAA-induced ALF rats on hCMEC/D3 cells were documented. Compared with serum from control rats, serum from APAP- or TAA-induced ALF rats showed remarkably higher arginase activity (Fig. 1A) and lower Arg levels (Fig. 1B). The culture medium containing 10% serum from the two ALF rats substantially impaired hCMEC/D3 viability (Fig. 1C, D), which could be reversed by the Arg supplementation (50 μg/mL) (Fig. 1C) or arginase inhibitor Nω-hydroxy-nor-Arg (nor-NOHA) treatment (50 μM) (Fig. 1D). Arg supplementation also almost abolished the hCMEC/D3 impairment induced by 20 μg/mL arginase (Fig. 1C). The above findings in hCMEC/D3 were further confirmed in rBMECs. Arg supplementation or nor-NOHA treatment also reversed rBMECs impairments induced by serum from the two ALF rats (Fig. 1E).

Our previous study demonstrated that Arg deficiency significantly inhibited the proliferation of hCMEC/D3 cells without affecting their apoptosis [8]. In the present study, we further found that the percentage of Sub-G1 cells in hCMEC/D3 cells treated with Arg-free medium ((-)Arg) was only about 1% and was not significantly different from that in the control group (Fig. S1A, B). But at the same time, Arg deficiency significantly increased the number of G1 phase cells whilst decreasing S phase cells (Fig. S1C). Similar results were also observed in rBMECs; Arg deficiency had no effect on the apoptosis of rBMECs (Fig. S1D). However, both Arg deficiency and arginase treatment significantly decreased EdU incorporated rBMECs (Fig. 1F) and delayed rBMECs in the G1 phase (Fig. 1G). The levels of key cell cycle regulatory proteins in the rBMECs were also measured. Both Arg deficiency and arginase treatment significantly downregulated the expression of Cyclin D, Cyclin-dependent kinase 2 (CDK2), and CDK4 in rBMECs but did not alter the expression of Cyclin A protein (Fig. 1H). These results suggest that ALF-induced Arg deficiency impaired brain microvascular endothelial cells primarily by disrupting the cell cycle.

### Involvement of NO-ROS and mTORC1-S6K1/4EBP1 pathway in impairment of hCMEC/D3 cells by Arg deficiency

Arg deficiency can trigger endothelial-type NO synthase uncoupling, leading to oxidative stress and endothelial dysfunction. Indeed, both Arg deprivation and arginase treatment significantly elevated ROS levels in hCMEC/D3 cells (Fig. S2A). However, the NO synthase inhibitor NG-nitro-L-arginine methyl ester showed only slight toxic effects on hCMEC/D3 cells (Fig. S2B). Neither the NO donor, sodium nitroprusside, nor the ROS scavenger, acetylcysteine, attenuated the cell impairment caused by Arg deprivation or arginase (Fig. S2C, D), indicating that the contribution of the NO-ROS pathway to hCMEC/D3 impairment by Arg deficiency was minor.

**Fig. 2:**
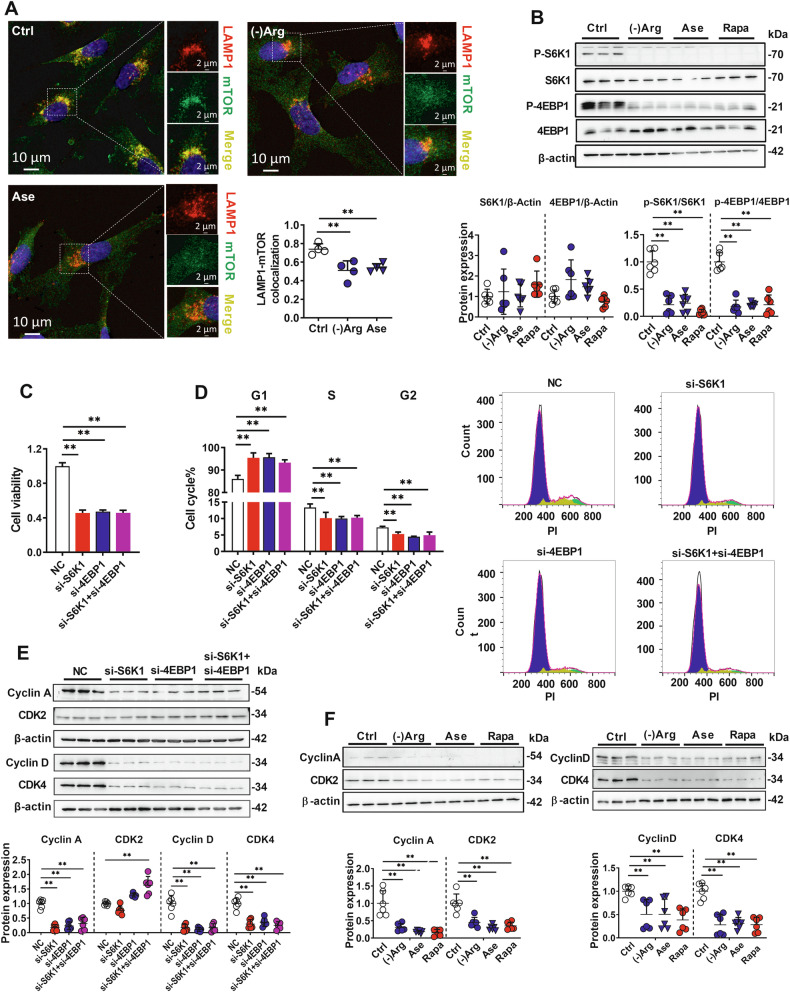
Involvement of mTORC1 in arginase-triggered hCMEC/D3 cells damage. **A** Co-immunofluorescence of mTOR (green) and LAMP1 (red) in hCMEC/D3 cells treated with arginine-free medium ((-)Arg) or 20 μg/mL arginase (Ase) (*n* = 4), calculated colocalization by Image-Pro Plus. **B** Phosphorylation of S6K1 and 4EBP1 in hCMEC/D3 cells treated with (-)Arg, Ase, or 20 nM rapamycin (Rapa) (*n* = 6). Cell viability (**C**) (*n* = 6) and cell cycle analysis (**D**) (*n* = 4) in hCMEC/D3 cells silencing S6K1 and 4EBP1. Cell cycle-related protein levels in hCMEC/D3 cells silencing S6K1 and 4EBP1 (**E**) or treated with (-)Arg, Ase, or Rapa (**F**) (n = 6). NC negative control. Data are expressed as mean ± SD. *p < 0.05; **p < 0.01. Statistical significance was determined with the 1-way ANOVA followed by the Dunnett post hoc test.

Arg also activates the mTORC1 pathway by promoting mTORC1 translocation to the lysosome surface and regulating S6K1 and 4EBP1 phosphorylation. We observed that the culture medium containing TAA rat serum substantially decreased the colocalization of mTOR and lysosomal-associated membrane protein 1 (LAMP1), and this effect of TAA serum could be partly reversed by Arg supplementation (Fig. S2E). Similarly, both Arg-free medium and arginase treatment also decreased the colocalization of mTOR and LAMP1 (Fig. 2A). Arg deficiency had no influence on LAMP1-positive lysosome levels (Fig. S2E and Fig. 2A). We also did not observe significant changes in the protein levels of TFEB and Galectin-3 under Arg-free medium treatment (Fig. S2F), which preliminarily ruled out the effects of lysosomal levels due to Arg deficiency. Furthermore, Arg deficiency, arginase, and the mTORC1 inhibitor Rapamycin significantly downregulated p-S6K1 and p-4EBP1 expression (Fig. 2B). These results proved that ALF-induced Arg deficiency could inhibit the mTORC1-S6K1/4EBP1 pathway. Involvement of S6K1 and 4EBP1 in cell cycle and proliferation was further investigated. Silencing S6K1 and 4EBP1 individually or both (Fig. S2G) significantly impaired hCMEC/D3 cell viability (Fig. 2C), arrested cells in the G1 phase (Fig. 2D), and decreased the expression of Cyclin A, Cyclin D, and CDK4 proteins in hCMEC/D3 cells (Fig. 2E). Similar to arginase treatment and Arg deficiency, rapamycin also impaired hCMEC/D3 cell viability (Fig. S4B), arrested cells in the G1 phase (Fig. S1C), and downregulated the expression of Cyclin A, Cyclin D, CDK2, and CDK4 (Fig. 2F).

### Insulin and silencing TSC2 reversed Arg deficiency-mediated mTORC1-S6K1/4EBP1 pathway inhibition and hCMEC/D3 cells impairments

**Fig. 3:**
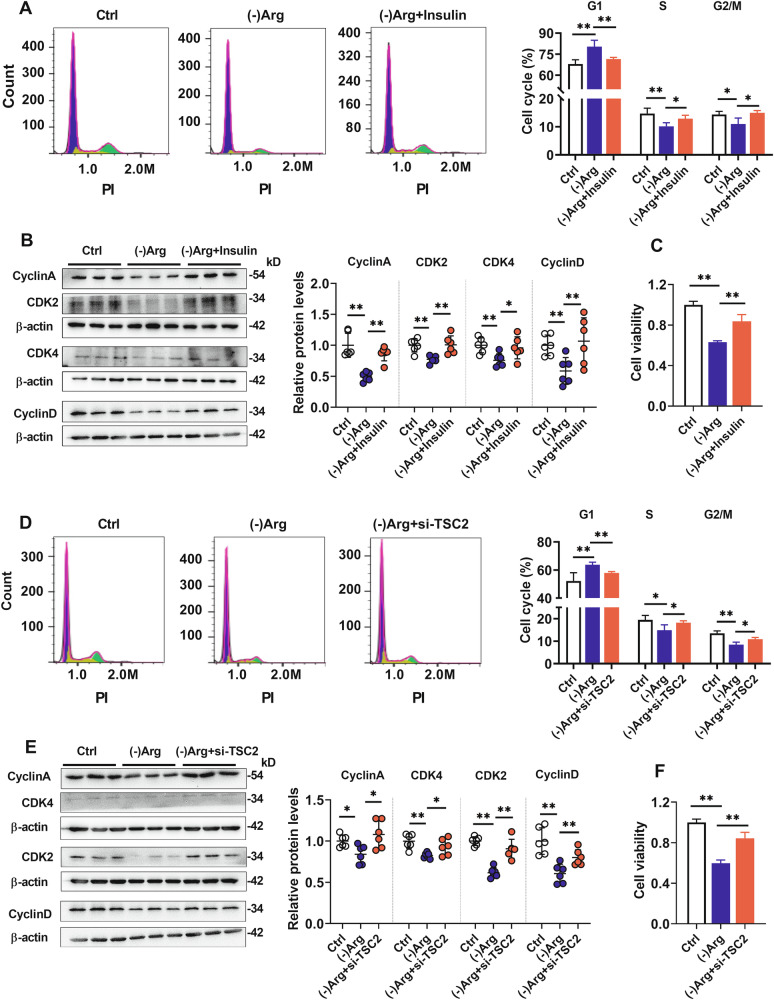
Inhibiting TSC2 attenuated Arg deficiency-mediated hCMEC/D3 cells damage. Cell cycle analysis on flow cytometer (*n* = 4) (**A**) and cell cycle-related protein expression (**B**) in hCMEC/D3 cells treated with (-)Arg and insulin (*n* = 6). **C** Effects of insulin on hCMEC/D3 cell damage caused by Arg deficiency (*n* = 6). Cell cycle analysis on flow cytometer (**D**) (*n* = 4) and cell cycle-related proteins expression (**E**) (*n* = 6) in hCMEC/D3 cells treated with (-)Arg and silencing TSC2. **F** Effects of silencing TSC2 on hCMEC/D3 cell damage caused by Arg deficiency (*n* = 6). Data are expressed as mean ± SD. *p < 0.05; **p < 0.01. Statistical significance was determined with the 1-way ANOVA followed by the Dunnett post hoc test.

mTORC1 can be suppressed by the localization of the tumor suppressor tuberous sclerosis complex 2 (TSC2) at the lysosome surface [35], and insulin can release mTORC1 from TSC2-mediated inhibition [36]. We found that insulin partly attenuated the Arg deficiency-mediated reduction of p-S6K1/S6K1 and p-4EBP1/4EBP1 (Fig. S3A) and the impairment of BBB cells (Fig. 3C). In line with this, insulin reversed the increases in fractions of the G1 phase (Fig. 3A) and downregulation in Cyclin A, Cyclin D, CDK2, and CDK4 proteins (Fig. 3B) induced by Arg deprivation. Silencing of TSC2 showed effects similar to those of insulin in Arg deficiency-mediated p-S6K1 and p-4EBP1 downregulation (Fig. S3B, C), hCMEC/D3 cell damage (Fig. 3F), and cell cycle alterations (Fig. 3D, E), further proving that mTORC1-S6K1/4EBP1 pathway suppression was involved in Arg deficiency-mediated hCMEC/D3 cell damage.

### Roles of autophagy in impairment of hCMEC/D3 cells by Arg deficiency

**Fig. 4:**
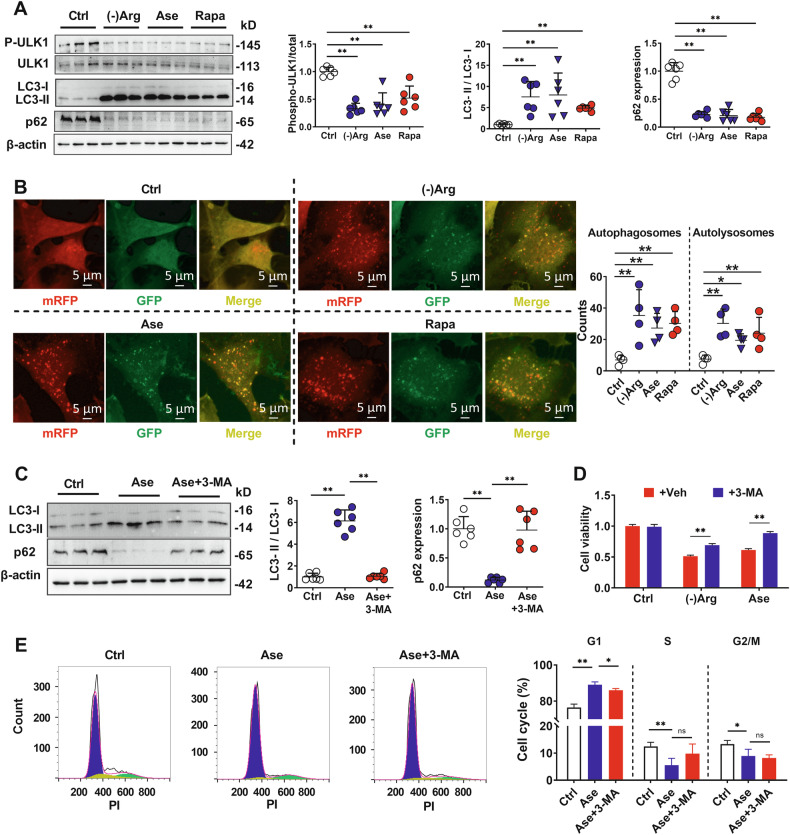
Involvement of autophagy in arginase-triggered hCMEC/D3 cell damage. **A** Protein levels of p-ULK1, LC3, and p62 in hCMEC/D3 cells treated with arginine-free medium ((-)Arg), 20 μg/mL arginase (Ase) or rapamycin (Rapa) (*n* = 6). **B** Autophagosomes (yellow) and autolysosomes (red) were monitored in hCMEC/D3 cells transfected with mRFP-GFP-LC3 adenoviral and treated with (-)Arg, Ase, or Rapa (*n* = 4). **C** Protein levels of LC3 and p62 in hCMEC/D3 cells treated with Ase combined with autophagy inhibitor 3-methyladenine (3-MA) (*n* = 6). Effect of 3-MA on hCMEC/D3 cell damage caused by (-)Arg or Ase (**D**) and on Ase-mediated hCMEC/D3 cell cycle arrest (**E**) (*n* = 4). Data are expressed as mean ± SD. *p < 0.05; **p < 0.01. Statistical significance was determined with the 1-way ANOVA or *t*-test.

mTORC1 modulates autophagy by phosphorylating Unc-51-like kinase 1 (ULK1) at Ser757 [37]. Similar to mTORC1 inhibitor rapamycin, both Arg deprivation and arginase treatment significantly decreased ULK1 phosphorylation, enhanced conversion from LC3-I to LC3-II (Fig. 4A), and increased the formation of autophagosomes (yellow dots) and autolysosomes (red dots) (Fig. 4B) in hCMEC/D3 cells, demonstrating autophagy induction. Consistently, significantly reduced p62 expression was also found in cells treated with rapamycin, Arg-free medium, and arginase, confirming autophagic flux completion (Fig. 4A).

We employed autophagy inhibitors 3-methyladenine and CQ to verify whether the endothelial cell damage caused by Arg deficiency is associated with autophagy activation. The results demonstrated that 3-methyladenine abolished the increased conversion of LC3-I to LC3-II and the decreased expression of p62 protein by arginase (Fig. 4C), and partly reversed arginase- or Arg deficiency-mediated hCMEC/D3 cell damage (Fig. 4D). In line with this, 3-methyladenine also partly attenuated cell cycle arrest by arginase (Fig. 4E) and reversed the arginase-mediated downregulation of Cyclin A and CDK2 proteins (Fig. S4A). Different from 3-methyladenine, CQ itself reduced the activity of hCMEC/D3 cells, while it had no obvious effect on the Arg deficiency-mediated cell damage and cycle arrest (Fig, S4B, C). This may be due to the indirect mTOR inhibition effects of CQ [38, 39], and autophagy-independent cytotoxic effects of CQ have also been found by other researchers [40].

**Fig. 5:**
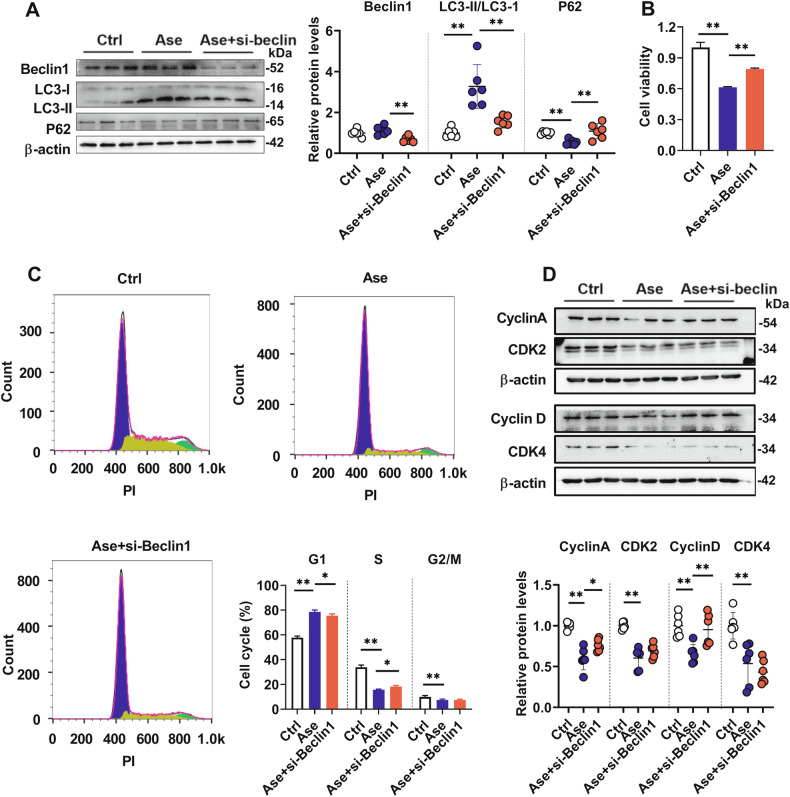
Silencing Beclin 1 partly reversed arginase-induced autophagy and cell cycle alterations. Effects of silencing Beclin1 (si-Beclin1) on Beclin1, LC3, and p62 protein levels in arginase (Ase) treated hCMEC/D3 cells (**A**) and on Ase-induced hCMEC/D3 cell damage (**B**) (*n* = 6). Cell cycle analysis on flow cytometer by PI staining (**C**) (*n* = 4) and cell cycle-related proteins expression in hCMEC/D3 cells (**D**) (*n* = 6) under Ase or Ase+si-Beclin1 treatment. Data are expressed as mean ± SD. *p < 0.05; **p < 0.01. Statistical significance was determined with the 1-way ANOVA followed by the Dunnett post hoc test.

BECLIN-1 is required for autophagosome formation [41]. The role of autophagy in hCMEC/D3 cell impairment by arginase was further confirmed by BECLIN-1 silencing (Fig. S4E). Similar to 3-methyladenine, silencing of BECLIN-1 significantly decreased the ratio of LC3-II to LC3-I, increased p62 protein levels (Fig. 5A), and partly attenuated the impairment of hCMEC/D3 cells induced by arginase treatment (Fig. 5B). Silencing of BECLIN-1 also slightly reversed cell cycle arrest in the G1 phase (Fig. 5C) and downregulation of Cyclin A and Cyclin D by arginase but unaffected CDK2 and CDK4 protein levels (Fig. 5D). These results suggested that mTORC1 suppression-mediated activation of autophagy is partially involved in Arg deficiency-mediated hCMEC/D3 cells damage.

### Involvement of Arg deficiency in BBB impairment in APAP- and TAA-induced ALF rats

**Fig. 6:**
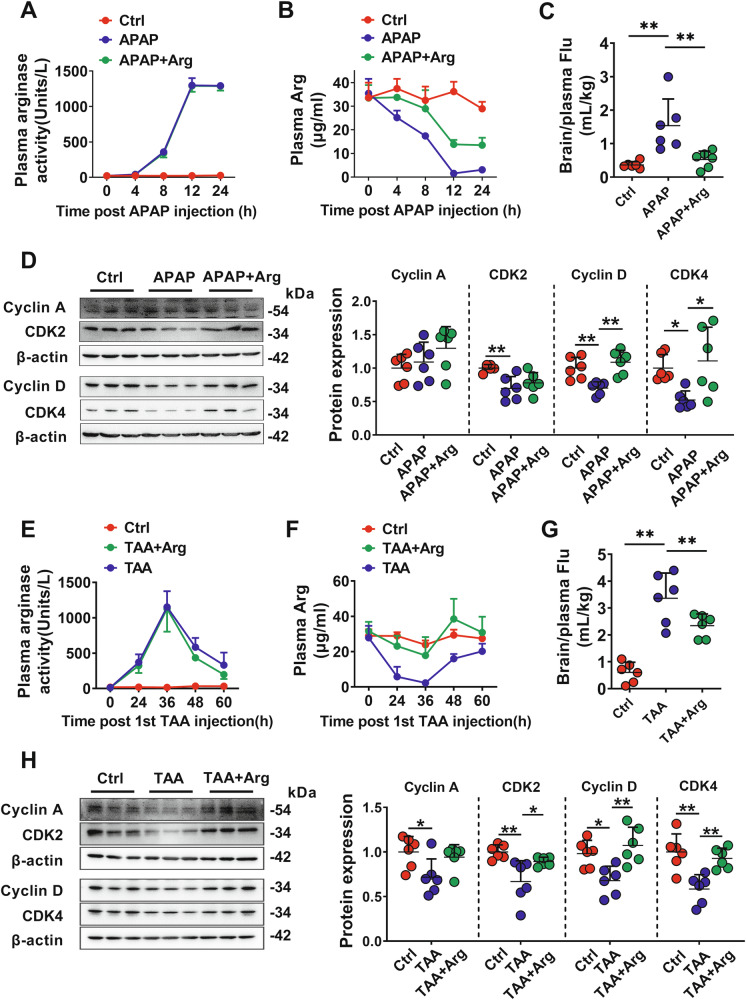
APAP- and TAA-induced ALF impaired BBB of rats due to substantially increased arginase activity. Plasma arginase activity (**A**), arginine (Arg) levels (**B**), and BBB permeability indexed by the ratio of brain fluorescein (Flu) to plasma (**C**) in control rats (Ctrl), rats intraperitoneally receiving acetaminophen (APAP rats), and APAP rats orally supplemented with 0.18 g/kg Arg (APAP+Arg rats) (*n* = 6). **D** Western blot of cell cycle-related proteins in isolated cerebral microvessels of Ctrl, APAP, and APAP+Arg rats (*n* = 6). Plasma arginase activity (**E**), Arg levels (**F**), and BBB permeability indexed (**G**) in Ctrl rats, rats intraperitoneally receiving thioacetamide (TAA rats), and TAA rats orally supplemented with 0.18 g/kg Arg (TAA+Arg rats) (*n* = 6). **H** Western blot of cell cycle-related proteins in isolated cerebral microvessels of Ctrl, TAA, and TAA+Arg rats (*n* = 6). Data are expressed as mean ± SD. *p < 0.05; **p < 0.01. Statistical significance was determined with the 1-way ANOVA followed by the Dunnett post hoc test.

To confirm that the substantial release of arginase from the injured liver impaired the BBB and its mechanism, APAP- and TAA-induced ALF rats were successfully established (Fig, S5). Both APAP- and TAA-induced ALF rats showed significantly higher plasma arginase activity (Fig. 6A, E) and lower Arg levels (Fig. 6B, F). The highest arginase activity (~1000 units/L) and lowest Arg level (near 1 μg/mL) were observed during 12 h following the APAP dose (Fig. 6A, B) or 36 h following the TAA dose (Fig. 6E, F).

ALF also significantly increased the penetration of fluorescein into the rat brain (Fig. 6C, G) and decreased rBMEC proliferation, as evidenced by the downregulation of biomarker proteins related to the cell cycle, Cyclin D, CDK2, and CDK4 (Fig. 6D, H). We also observed decreased phosphorylation levels of S6K1, 4EBP1, and ULK1, an enhanced ratio of LC3-Ⅱ to LC3-Ⅰ, and decreased expression of p62 protein in the cerebral microvessels of APAP- (Fig. S6A, B) and TAA-induced ALF rats (Fig. S6C, D), demonstrating the inhibition of mTORC1-S6K1/4EBP1 and induction of autophagy in these ALF rats. More importantly, oral Arg supplementation significantly attenuated most alterations in the brains of APAP- and TAA-induced ALF rats (Fig. 6 and Fig. S6) without improving liver function (Fig. S5).

### Effects of arginase treatment on BBB in rats

**Fig. 7:**
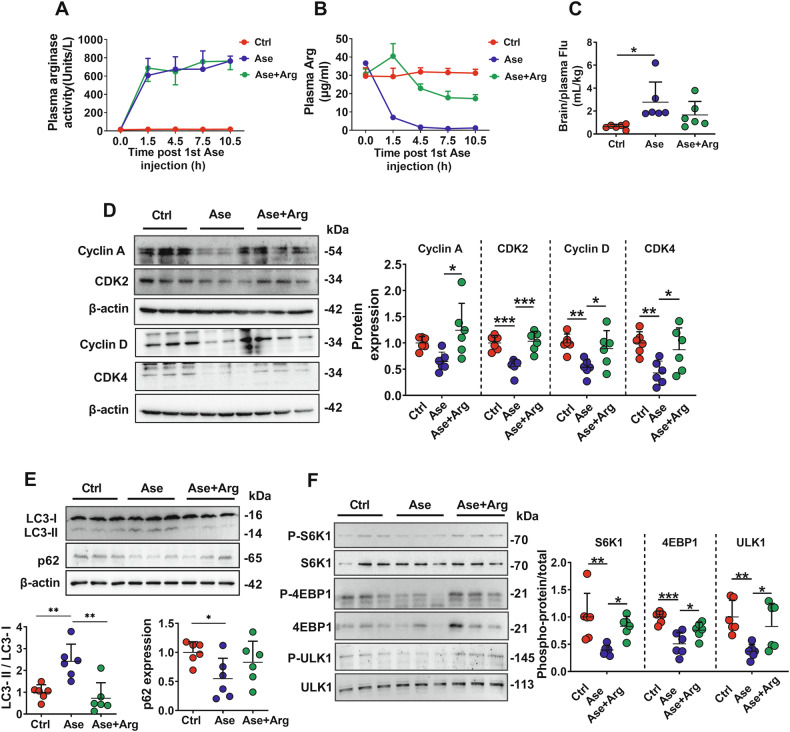
Arginase impaired BBB of rats via depriving Arg. Arginase activity (**A**) and arginine (Arg) levels (**B**) in plasma of control rats (Ctrl), rats receiving multiple-dose arginase (71.25 Units/kg) intravenously (Ase rats) and Ase rats orally supplemented with 0.18 g/kg Arg (Ase+Arg rats) (*n* = 6). **C** BBB permeability indexed by the ratio of brain fluorescein (Flu) to plasma in experimental rats (*n* = 6). Western blot of cell cycle-related proteins (**D**), autophagy markers (LC3 and p62) (**E**), and mTORC1 signal pathway-related proteins (S6K1, 4EBP1, and ULK1) phosphorylation (**F**) in isolated cerebral microvessels of experimental rats (*n* = 6). Data are expressed as mean ± SD. *p < 0.05; **p < 0.01. Statistical significance was determined with the 1-way ANOVA followed by the Dunnett post hoc test.

To confirm the role of excessive arginase in BBB impairment, rats received multi-doses of arginase. Plasma arginase activity in arginase-treated rats was maintained over 600 units/L (Fig. 7A), and Arg levels decreased to less than 2 μg/mL (Fig. 7B) during arginase treatment. A striking increase in the distribution of fluorescein in the brains of arginase-treated rats (Fig. 7C) indicated BBB impairment. Arginase treatment significantly decreased the expression of biomarker proteins related to the cell cycle (Fig. 7D), demonstrating inhibition of rBMEC proliferation. Furthermore, inhibition of mTORC1-S6K1/4EBP1 and induction of the autophagy pathway were also observed in arginase-treated rats (Fig. 7E, F). Similar to the results in ALF rats, Arg supplementation also efficiently reversed most of the alterations in rats treated with arginase (Fig. 7).

## Discussion

Hepatic encephalopathy is a severe neuropsychiatric syndrome caused by liver failure with the involvement of BBB breakdown of which the mechanism remains unclear. Much research has focused on increased circulating toxins due to the deterioration of liver function, whereas less attention has been paid to detrimental molecules released from the injured liver. We observed that ALF, whether induced by hepatic ischemia-reperfusion [8], APAP, or TAA, leads to a substantial release of arginase into the serum, resulting in Arg deficiency and ultimately damaging the BBB. Similar increases in arginase activity have also been observed in patients with acute liver cirrhosis, alcoholic liver disease, and liver cancer [12–14], conditions often associated with BBB damage and hepatic encephalopathy. In vitro data showed that both the arginase inhibitor nor-NOHA and Arg supplementation mitigated the impairment of hCMEC/D3 and rBMECs by ALF rat serum, arginase, and Arg deficiency, suggesting that ALF impairs the BBB mainly by depriving Arg due to increased plasma arginase activity.

Arg is crucial for the maintenance of normal physiological activity. Arg deficiency can induce immunosuppression [42] and impair glucose uptake [43], but its role in BBB impairment has rarely been reported. The present study revealed that both Arg deficiency and arginase accumulation impaired BBB cells by inhibiting cell proliferation, retarding cells in the G1 phase, and downregulating the protein expression of CDK2, Cyclin D, and CDK4. Most Cyclins and CDKs are essential for driving the cell cycle in mammals. CDK4 knockdown induces cytoplasmic retention of Cyclin D and G0/G1 phase arrest of the HeLa cells [44]. Autophagy-mediated protein degradation of Cyclin D also induces G1 cell cycle arrest in breast cancer cells [45]. But researchers have revealed that Cdk2^-/-^ mice are viable and survive for up to 2 years, indicating that CDK2 is dispensable for proliferation and survival of most cell types [46, 47]. These results suggest that Arg deficiency-mediated downregulation of CDK4 and Cyclin D mainly contributes to the inhibition of BBB cell proliferation. It is worth noting that Arg deficiency and arginase had little effect on the expression of Cyclin A in rBMECs, and the decreased extent of CDK2, Cyclin D, and CDK4 proteins in rBMECs was less than that in hCMEC/D3 cells, which may partly explain why the toxic effects of ALF rat serum on rBMECs were less than those on hCMEC/D3 cells and why the expression of Cyclin A in APAP-induced ALF rats and arginase-treated rats was unaltered.

Arg promotes the translocation of mTOR to the lysosomal surface and regulates the phosphorylation of S6K1 and 4EBP1 [24], and cell cycle progression is highly controlled by mTORC1 via stimulating protein synthesis through phosphorylating S6K1 and 4EBP1 [48, 49]. In hCMEC/D3 cells, we observed that both TAA rat serum and Arg-free medium substantially decreased the colocalization of mTOR and LAMP1-labeled lysosomes, whilst not influencing the levels of LAMP1-positive lysosomes. Meanwhile, we also did not observe significant changes in the protein levels of TFEB and Galectin-3 under Arg-free medium treatment, which preliminarily ruled out the effects of lysosome biogenesis and lysosomal membrane damage by Arg deficiency. These results prove that ALF-induced Arg deficiency can inhibit mTORC1 activation in hCMEC/D3 cells, and this effect is independent of changes in lysosome levels. Arg deficiency-mediated mTORC1 signaling inhibition could downregulate p-S6K1 and p-4EBP1 protein expression. Silencing of S6K1 or 4EBP1 showed effects similar to those of Arg deficiency on the cell cycle and related protein expression, indicating that Arg deficiency impairs BBB cells by retarding cells in the G1 phase through inhibition of the mTORC1-S6K1/4EBP1 pathway.

Insulin stimulates PI3K-AKT-mTORC1 signaling primarily by controlling the localization of TSC 1/2 to the lysosome surface [35, 36], and TSC2 is a negative regulator of mTORC1 signaling [35]. We found that both insulin treatment and silencing TSC2 nearly neutralized the Arg deficiency-mediated downregulation of p-S6K1 and p-4EBP1 proteins, impairment of hCMEC/D3, and changes in cell cycle, further supporting the key roles of the mTORC1-S6K1/4EBP1 pathway in Arg deficiency-mediated BBB cell damage. In addition, Arg could increase the AMPK expression and activity to modulate energy balance [50], and AMPK triggers activation of TSC2 by direct phosphorylation on T1271 and S1387 [51]. Mutating these AMPK sites on TSC2 partially counteracted the reduction in mTORC1 substrate S6K1 and 4EBP1 phosphorylation, following AMPK activation by glycolysis inhibitor 2-deoxyglucose [51]. These results suggest that ALF-induced Arg deficiency may affect the AMPK-mTORC1 pathway to modulate energy metabolism, which may also be related to BBB damage in ALF animals. However, we found that Arg deficiency had no effect on AMPK expression or phosphorylation in hCMEC/D3 cells (Fig. S7A), and ALF showed no obvious influence on rats’ fasting blood glucose levels (Fig. S5E, K), suggesting that Arg deficiency-mediated mTORC1 inhibition in BBB cells may be unrelated to the AMPK pathway.

mTORC1 inhibition also induces autophagy by inhibiting the phosphorylation of ULK1 at Ser757 [37]. We found that Arg deficiency induced autophagy in hCMEC/D3 cells. Normally, autophagy helps clear damaged proteins from cells, but hyperactive autophagy often leads to cellular dysfunction and death under pathological conditions [26, 27, 52]. The role of autophagy activation in BBB impairment remains controversial. Yang et al. found that autophagy alleviated hypoxia-induced BBB injury by regulating claudin-5 expression [53], whilst other researchers revealed that cerebral ischemia-induced [26] or HIV Tat-mediated [27] autophagy activation disrupts BBB function by decreasing tight junction protein expression in brain endothelial cells. The present study showed that inhibition of autophagy with 3-methyladenine or silencing Beclin-1 partially abrogated Arg deficiency-mediated cell impairments and alterations in cell cycle-related proteins, indicating that excessive autophagy activation also partially contributes to Arg deficiency-induced BBB cell cycle arrest. Autophagy induction is involved in the degradation of CDK2 [54] and cyclin D1 [45]. AMBRA1 (activating molecule in beclin-1-regulated autophagy) regulates the abundance of D-type cyclins by mediating their degradation to control the transition from G1 to S phase [55]. These results can also support our findings.

Interestingly, unlike 3-MA or Beclin-1 silencing, another classic autophagy inhibitor CQ did not significantly reverse Arg deficiency-mediated alterations in cell cycle. This phenomenon may be attributed to CQ’s unique mechanism: CQ blocks autophagic flux by impairing lysosomal function [56], which concurrently interferes with the activation platform of mTORC1 on the lysosomal membrane [57]. Recent studies have reported this indirect mTORC1 inhibition effect of CQ [38, 39]. Given our finding that Arg deficiency also directly inhibits the mTORC1-S6K1/4EBP1 pathway to affect cell cycle progression, CQ’s impact on cell cycle via autophagy inhibition may be counteracted by its indirect suppression of mTORC1. These findings further elucidate that Arg deficiency-induced BBB cell damage and cell cycle alterations result from both inhibition of the mTORC1-S6K1/4EBP1 pathway and induction of autophagy. It is reported that S6K1 and 4EBP1 are involved in autophagy induction [58, 59]. However, our results demonstrated that silencing S6K1 or 4EBP1 did not affect LC3-I to LC3-II conversion or the expression of p62 and BECLIN-1 proteins (Fig. S7B). The autophagy inhibitor 3-methyladenine did not attenuate BBB impairment caused by silencing S6K1 or 4EBP1 (Fig. S7C). The association between autophagy induction and S6K1 and 4EBP1 inhibition in BBB cells requires further investigation.

The roles of increased arginase activity and decreased Arg levels in BBB breakdown were further documented in APAP- and TAA-induced ALF rats, and arginase-treated rats. Significantly lower expression of biomarker proteins related to the cell cycle, inhibition of the mTORC1-S6K1/4EBP1 pathway, and induction of autophagy were observed in the cerebral microvessels of the three experimental rats. Arg supplementation attenuated most alterations in the brains of the ALF rats and arginase-treated rats. All these results further support our hypothesis that ALF leads to substantial arginase release from the injured liver, depriving plasma Arg to impair the BBB by retarding cells in the G1 phase due to both inhibition of the mTORC1-S6K1/4EBP1 pathway and induction of the mTORC1-autophagy pathway.

The present study emphasizes the importance of ALF-induced Arg deficiency in cerebrovascular endothelial cell damage and provides new insights into the prevention and treatment of ALF-induced BBB dysfunction. It is worth noting that although the change in circulation Arg levels has direct effects on endothelial cells, Arg deficiency-mediated BBB damage is still likely a comprehensive result of multiple factors and cells, and the impairments of other BBB component cells or neuroinflammation may also be involved. We have demonstrated that the effects of Arg deficiency on the NO-ROS pathway do not contribute to the regulation of endothelial cell damage. Similar NO-ROS-independent effects of Arg have also been reported in other studies [60, 61], which can further support our findings. However, NO itself is a key signaling molecule that regulates vasodilation and maintains BBB permeability. A reduction in NO can lead to vasoconstriction and hemodynamic abnormalities [62], which may indirectly cause BBB damage. Furthermore, Arg modulates microglial polarization, promoting the anti-inflammatory M2 phenotype and suppressing the pro-inflammatory M1 phenotype [63], indicating that Arg deficiency may shift microglia toward M1 dominance, amplifying neuroinflammation and impairing BBB function. These effects of Arg deficiency may also be one of the reasons for BBB damage, which still needs further investigation.

## Materials and methods

### Chemicals and reagents

Chemicals and reagents were listed in the Supplementary Tables S1–S4.

### Animals

Male Sprague-Dawley rats (aged 8–9 weeks, weighing 220–250 g), from SINO-BRITISH SIPPR/BK Lab. ANIMAL Co., Ltd (Shanghai, China), were housed at constant room temperature and humidity on a normal 12 h light/dark cycle with free access to laboratory food and water. All animal studies were performed in accordance with the Guide for the Care and Use of Laboratory Animals (National Institutes of Health) and approved by the Animal Ethics Committee of China Pharmaceutical University.

### ALF animal models

TAA- and APAP-induced liver failure rats were established as previously reported [5, 64]. After the ALF models were successfully developed, the rats were killed under isoflurane anesthesia, and serum or brain samples were obtained. Arginase activity and Arg levels in rat serum were measured using an arginase activity kit and an HPLC method, respectively, as described previously [8].

### BBB cell culture, drug treatment, and cell viability assay

Both hCMEC/D3 cells and primary cultured rBMECs were used as in vitro BBB models. hCMEC/D3 cells was purchased from FuHeng Cell Center (FH1110, STR fingerprinting) and cultured in RPMI-1640 medium. Primary rBMECs were isolated and cultured in DMEM/F-12 according to our previously reported method [65]. These BBB cells were seeded in 96 wells at a density of 3 × 10^5^ cells/mL. Following confluence (~80%), the cells were incubated with mediums containing serum of TAA- or APAP-induced ALF rats or 20 μg/mL arginase with or without 50 μM arginase inhibitor nor-NOHA or 50 μg/mL Arg supplement for 24 h. The rat serum content used in this study (10% for hCMEC/D3 and 50% for rBMECs) was designed according to pre-experiments. These BBB cells were also incubated in an Arg-free medium or medium containing the other tested agents. Subsequently, cell viability was assessed using CCK-8 kits; cell proliferation and cell cycle analysis were performed using kits YF®488 Click-iT EdU Kit and Cell Cycle Detection Kit, respectively, according to their instructions on a MACS Quant flow cytometer (Miltenyi Biotec, Germany). The data were analyzed using FlowJo 10.4 software. The expression of cell cycle-related proteins was measured simultaneously using western blotting.

### Small interfering RNA transfection

hCMEC/D3 cells were transfected with 100 nmol/L small interfering RNA targeting *S6K1*, *4EBP1*, *BECLIN-1*, or *TSC2* (whose sequences in Table S2) using Lipofectamine 3000 in an antibiotic-free medium. Control cells were transfected with a negative control siRNA. After 6 h of transfection, the medium was replaced with normal medium. Following 24 h incubation, the transfected cells were incubated with medium containing the test agents for another 24 h for western blotting or cell cycle analysis.

### mRFP-GFP-LC3 transfection

hCMEC/D3 cells were seeded in 24-well plates and transfected with 1 × 10^7^ PFU/well mRFP-GFP-LC3 adenoviral vectors. Following 8 h of transfection, the cells were treated with Arg-free medium or arginases for 24 h. Then the cells were fixed in 4% paraformaldehyde. The LC3 puncta were observed using a Zeiss LSM700 confocal microscope (Zeiss, Germany). Yellow (mRFP+ GFP+) and red puncta (mRFP+ GFP-)indicate autophagosomes and autolysosomes, respectively.

### Effects of APAP-induced ALF on BBB integrity of rats

Eighteen rats were randomly divided into the control, APAP, and APAP+Arg groups. Control rats received normal saline. APAP rats were i.p. administered 500 mg/kg APAP. For APAP+Arg rats, APAP rats orally received Arg (0.18 g/kg) before and at 12 h following the APAP dose. Plasma arginase activity and Arg levels were monitored during ALF development. At 24 h post-APAP administration, the rats were i.v. injected fluorescein (2 mg/kg). After 45 min, the rats were sacrificed, and liver and blood samples were collected. Cerebral cortices were quickly obtained to measure fluorescein concentration and perform immunofluorescence analysis. Cerebral microvessels were isolated to measure the protein expression of related biomarkers. Liver injury was confirmed using serum biochemical parameters and histological examination as we described [66].

### Effects of TAA-induced ALF on BBB integrity of rats

Eighteen rats were randomly divided into control, TAA, and TAA+Arg groups. Control rats received normal saline vehicles. The TAA rats received i.p. TAA (300 mg/kg) daily for 3 consecutive doses. TAA+Arg rats and TAA rats were orally administered Arg (0.18 g/kg twice daily) or vehicle, respectively. Plasma arginase activity and Arg levels were monitored during ALF development. Twelve hours after the last dose of TAA, BBB function, corresponding biomarkers, and liver function were measured, as described for APAP-induced ALF rats.

### Effects of arginase on BBB integrity of rats

Crude arginase (9.5 units/mg protein) from rat liver was prepared and purified as previously reported [8]. Eighteen rats were randomly divided into control, arginase-treated (Ase), and arginase-treated rats supplied with Arg (Ase+Arg) groups. Control rats received normal saline. The pre-experiment demonstrated that 5 min following the intravenous injection of arginase (71.25 units/kg) into rats, plasma arginase activity increased to over 1500 units/L, then quickly decreased to normal levels at 120 min (Fig. S8B). This increase in arginase activity corresponded with a significant reduction in plasma levels of Arg, which dropped from 35 μg/mL prior to the arginase injection to ~1 μg/mL, lasting for 90 min (Fig. S8A). Based on these data, Ase rats were i.v. administered arginase (71.25 units/kg) via the tail vein at intervals of 1.5 h for seven doses. Ase+Arg rats orally received Arg (0.18 g/kg) before the first dose of arginase. Plasma arginase activity and Arg levels were monitored during arginase treatment. At 0.5 h after the last arginase dose, BBB function and the corresponding biomarkers were measured as described for APAP-induced ALF rats.

### Immunofluorescence assay

For hCMEC/D3 cells, the cultured cells on coverslips were fixed in 4% paraformaldehyde, blocked in 5% goat serum containing Triton X-100, and incubated with mouse anti-LAMP1 and rabbit anti-mTOR antibodies at 4 °C overnight, followed by incubation with corresponding secondary antibodies. Localization of mTOR (red) in the lysosomes of hCMEC/D3 cells was visualized by co-localization of mTOR (red) with LAMP1 (green), and the overlap coefficient R of red-green was calculated using Image-Pro Plus 6.0.

### Western blot

Protein samples were extracted from cells and tissues and electrophoresed on SDS-PAGE, then transferred to polyvinylidene fluoride membranes as we described [67]. Following blocking, the membrane was incubated with corresponding primary antibodies overnight at 4 °C followed by secondary antibodies for another 2 h at room temperature. Immunoreactive bands were visualized with HRP substrate (Vazyme Biotech, Nanjing, China) using the 5200 Multi Chemiluminescent Imaging System (Tanon Technology, Shanghai, China).

### Statistical analysis

All values are expressed as mean ± SD. Normality was assessed using Shapiro-Wilk’s normality test. To compare two sample groups, the Unpaired 2-tailed *t*-test was used. For multiple groups, statistical significance was determined with the one-way ANOVA followed by Dunnett’s post hoc test. Statistical analysis was carried out using Graphpad Prism 8.0.2. *p* ≤ 0.05 is considered to indicate a statistical difference. Sample size was determined based on similar studies in this field, and there were no data exclusions.

Supplementary information is available at (Cell Death & Disease)’s website.

## Supplementary information

Original Figures

Extended Data Figures and Tables

## Data Availability

The datasets generated during and/or analyzed during the current study are available in the supplementary files accompanying this manuscript.
