# Supplementary material for: Acute liver failure-induced arginine deficiency impairs blood-brain barrier via inhibiting mTORC1-S6K1/4EBP1 pathway and inducing autophagy
Source: Cell Death Dis. 2025 Nov 17;16(1):842. doi: 10.1038/s41419-025-08152-4 (PMC12623816; doi:10.1038/s41419-025-08152-4)
Supplement: Supplementary file 1 — Original Figures [file 41419_2025_8152_MOESM1_ESM.pdf]

Figure1 H:

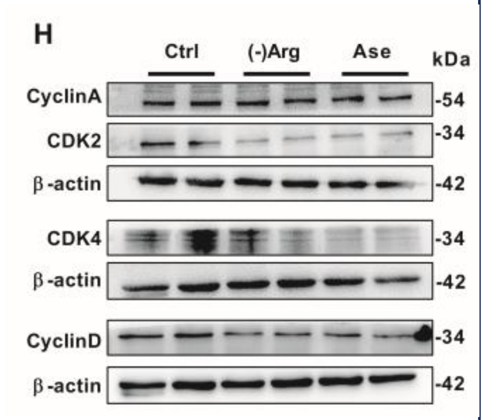

H
Ctrl
(-)Arg
Ase
kDa
CyclinA
-54
CDK2
-34
β-actin
-42
CDK4
-34
β-actin
-42
CyclinD
-34
β-actin
-42

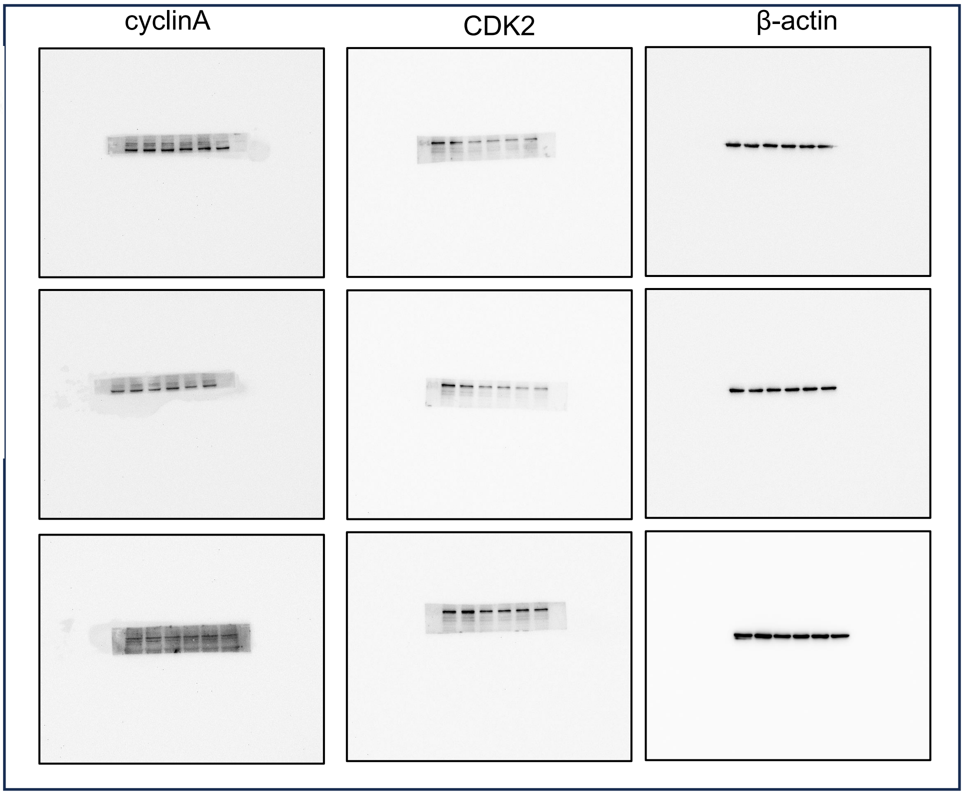

cyclinA
CDK2
β-actin

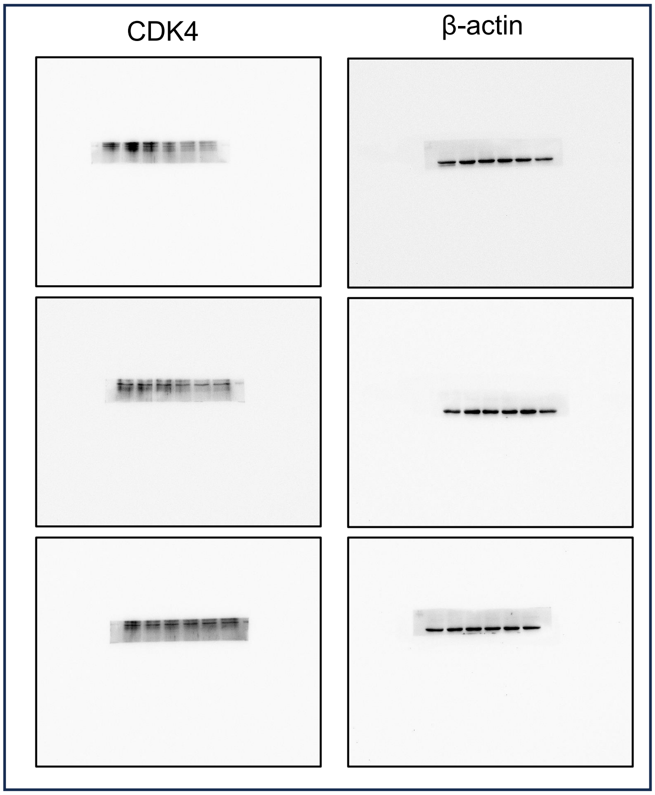

CDK4
β-actin

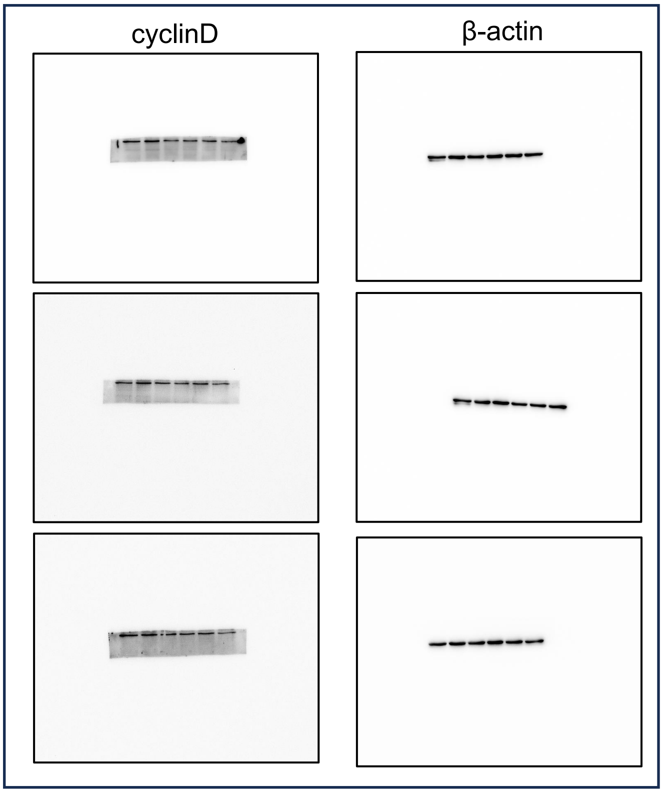

cyclinD
β-actin

Figure2 A

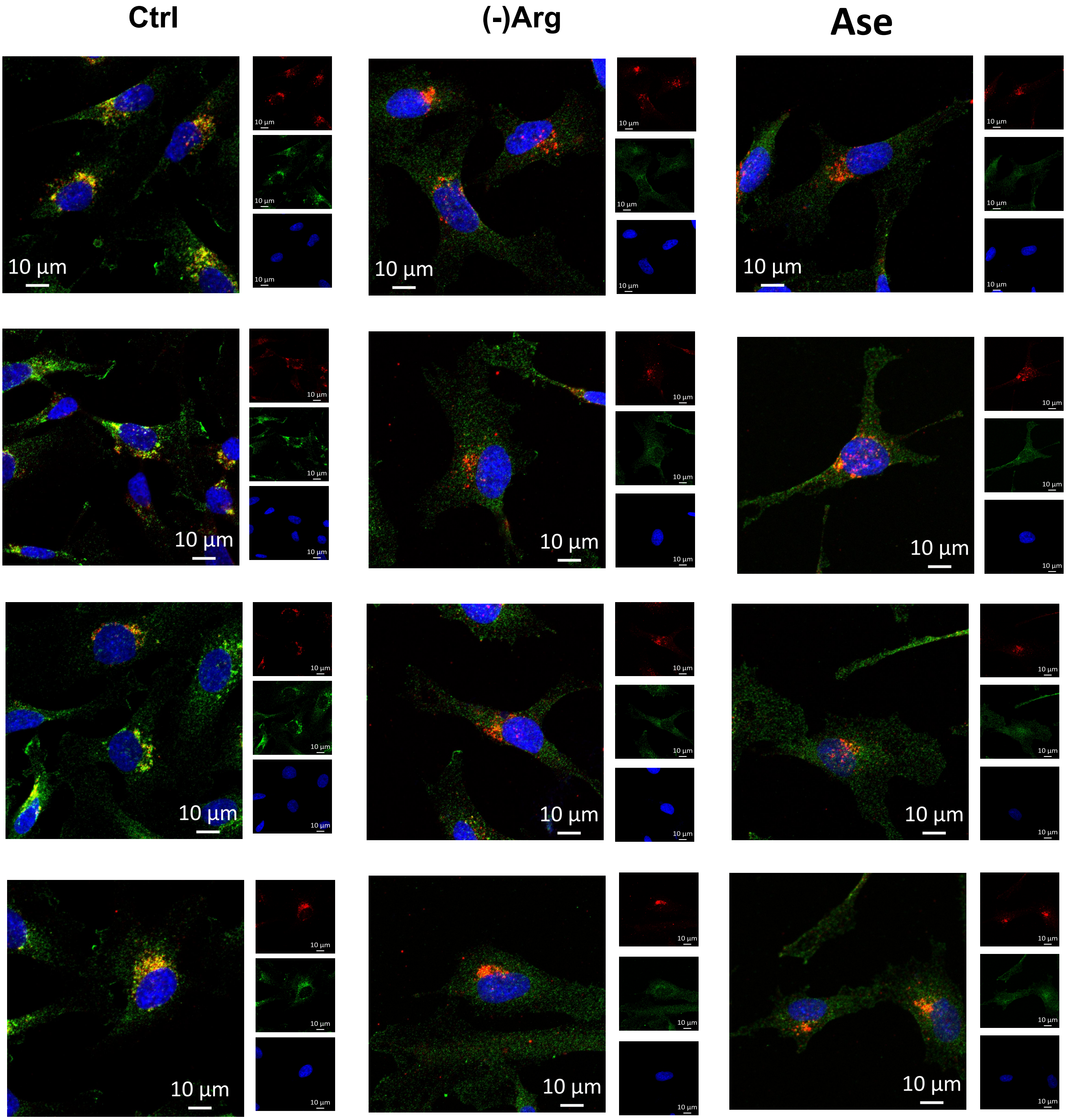

Figure2 B:

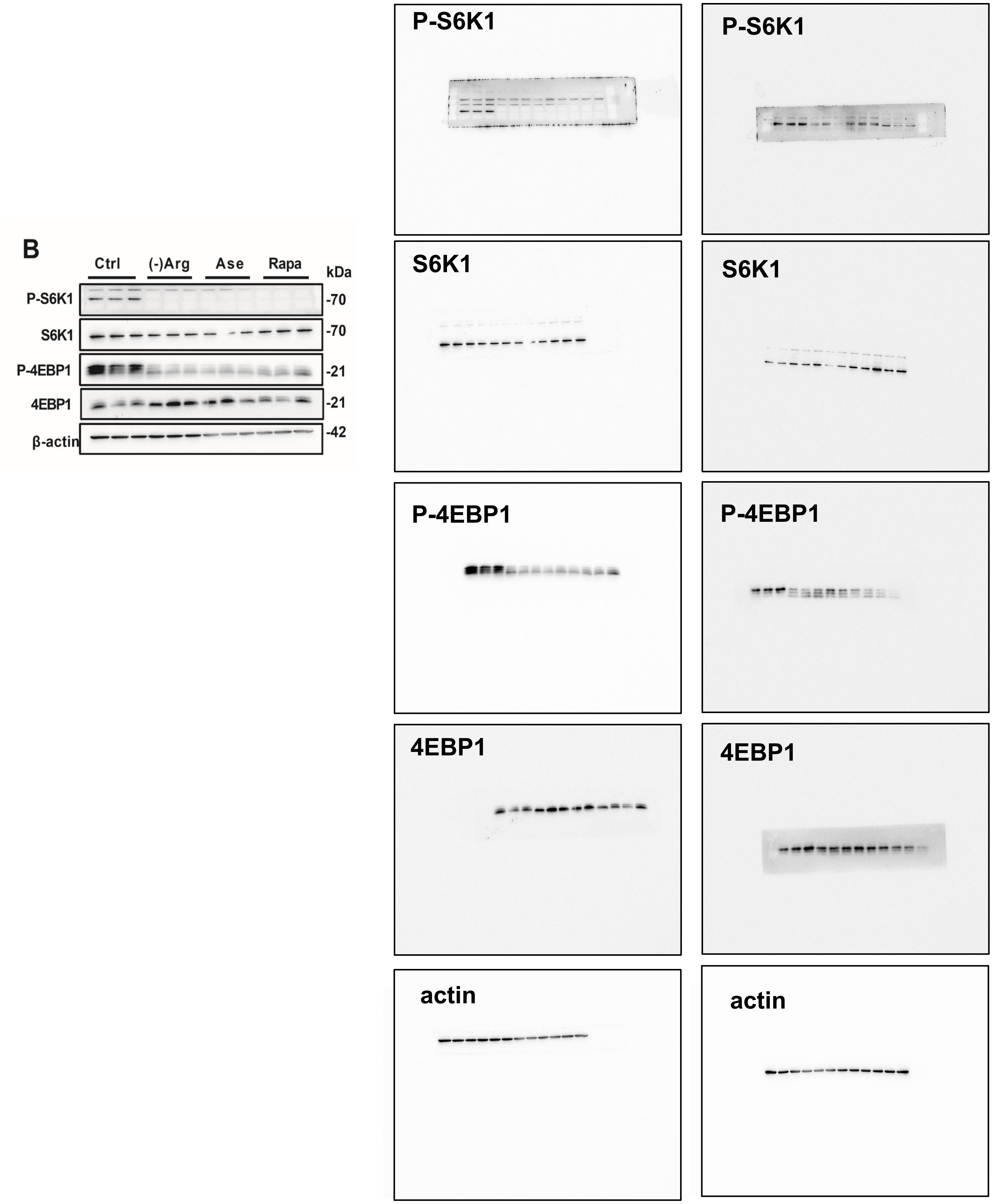
B
Ctrl
(-)Arg
Ase
Rapa
kDa
P-S6K1
-70
S6K1
-70
P-4EBP1
-21
4EBP1
-21
β-actin
-42
P-S6K1
P-S6K1
S6K1
S6K1
P-4EBP1
P-4EBP1
4EBP1
4EBP1
actin
actin

Figure2 E:

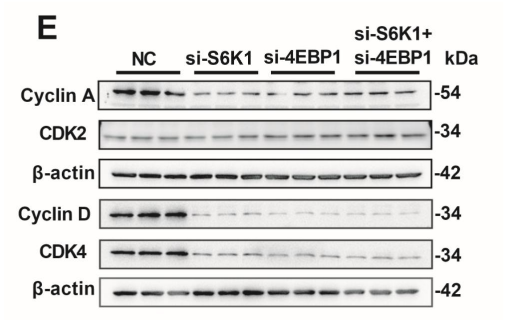
E
NC
si-S6K1
si-4EBP1
si-S6K1+
si-4EBP1
kDa
Cyclin A
-54
CDK2
-34
β-actin
-42
Cyclin D
-34
CDK4
-34
β-actin
-42

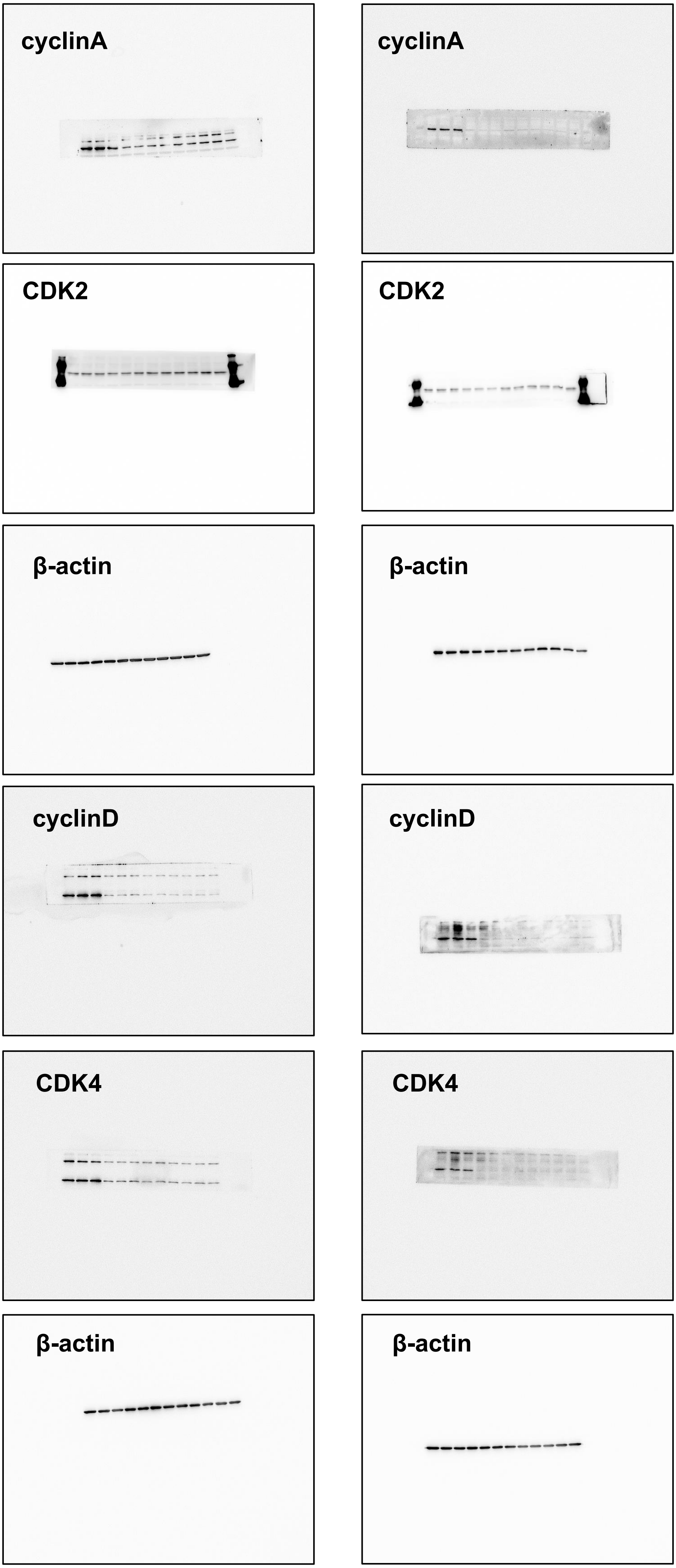
cyclinA
cyclinA
CDK2
CDK2
β-actin
β-actin
cyclinD
cyclinD
CDK4
CDK4
β-actin
β-actin

Figure2 F:

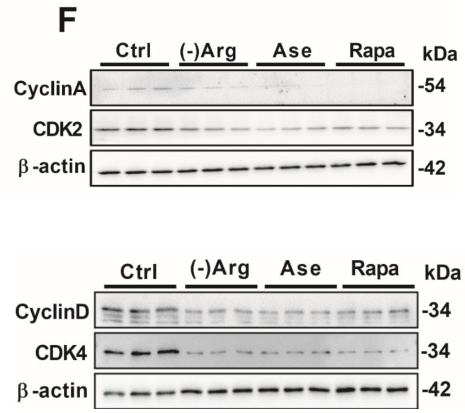
F
Ctrl
(-)Arg
Ase
Rapa
kDa
CyclinA
-54
CDK2
-34
β-actin
-42
Ctrl
(-)Arg
Ase
Rapa
kDa
CyclinD
-34
CDK4
-34
β-actin
-42

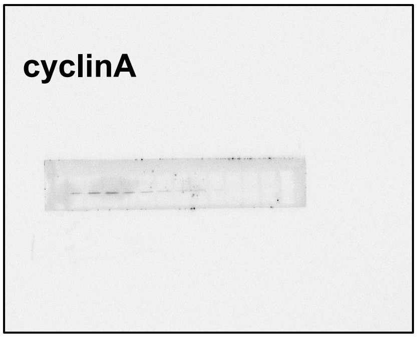
cyclinA

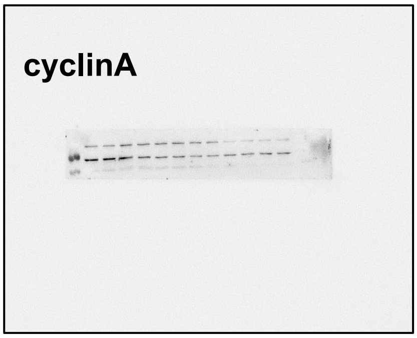
cyclinA

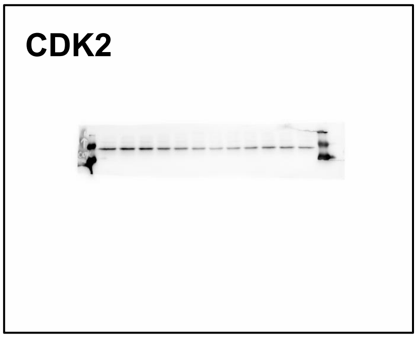
CDK2

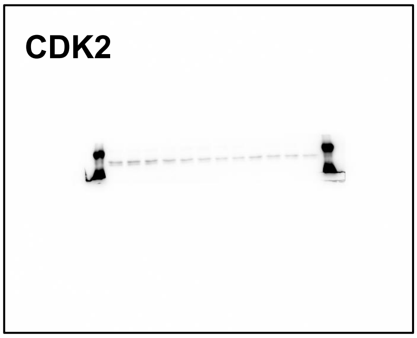
CDK2

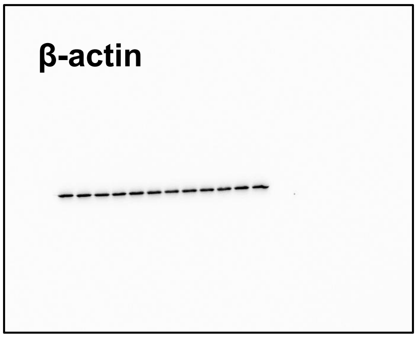
β-actin

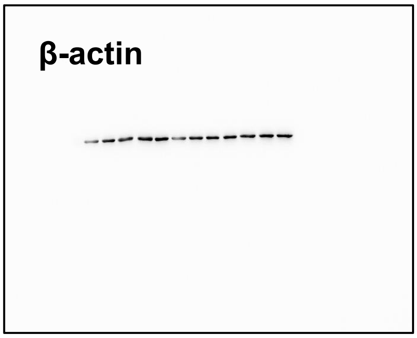
β-actin

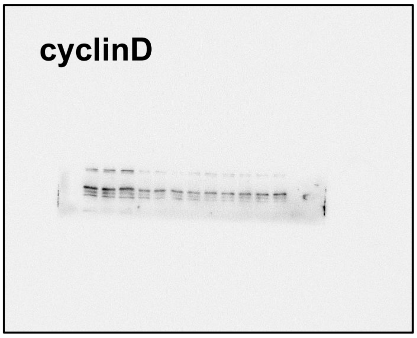
cyclinD

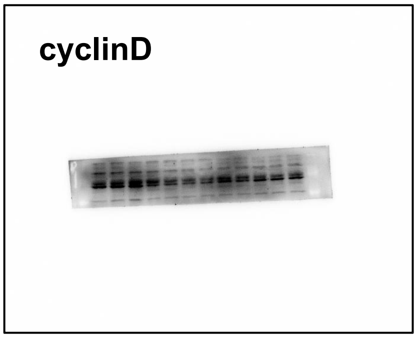
cyclinD

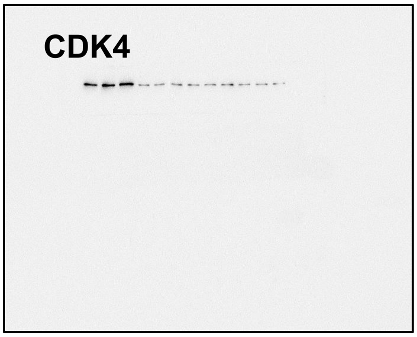
CDK4

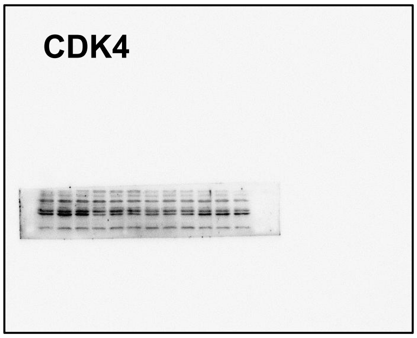
CDK4

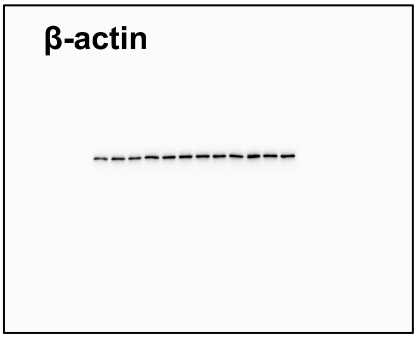
β-actin

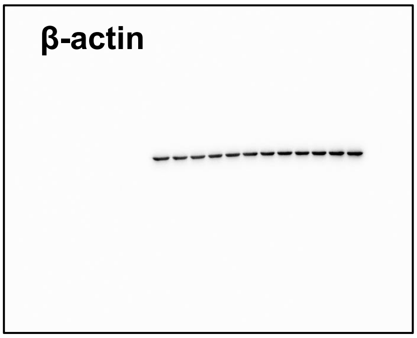
β-actin

Figure3 B:

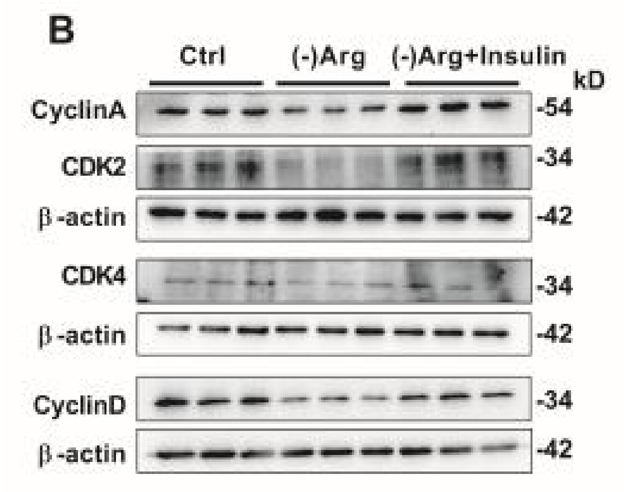

B
Ctrl
(-)Arg
(-)Arg+Insulin
kD
CyclinA
-54
CDK2
-34
β-actin
-42
CDK4
-34
β-actin
-42
CyclinD
-34
β-actin
-42

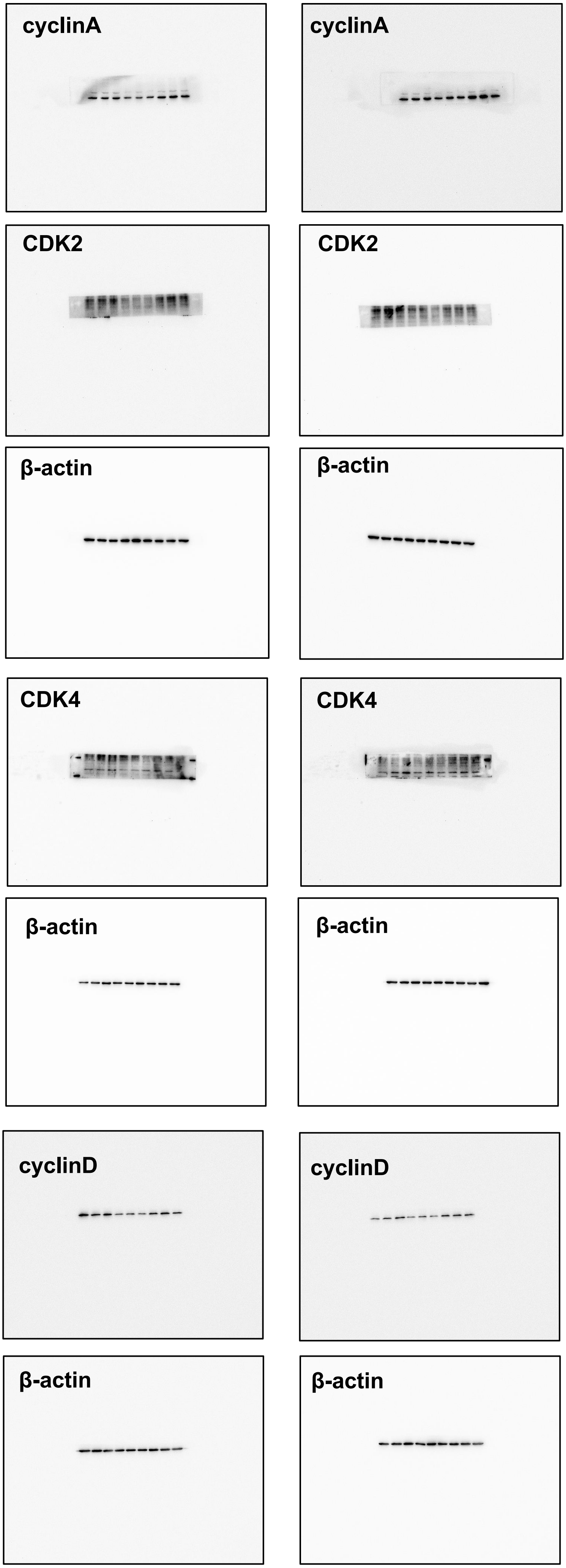

cyclinA
cyclinA
CDK2
CDK2
β-actin
β-actin
CDK4
CDK4
β-actin
β-actin
cyclinD
cyclinD
β-actin
β-actin

Figure3 E:

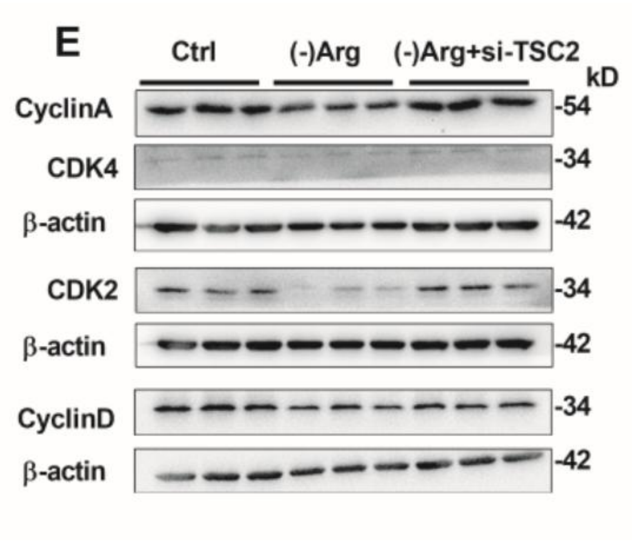
E
Ctrl
(-)Arg
(-)Arg+si-TSC2
kD
CyclinA
-54
CDK4
-34
β-actin
-42
CDK2
-34
β-actin
-42
CyclinD
-34
β-actin
-42

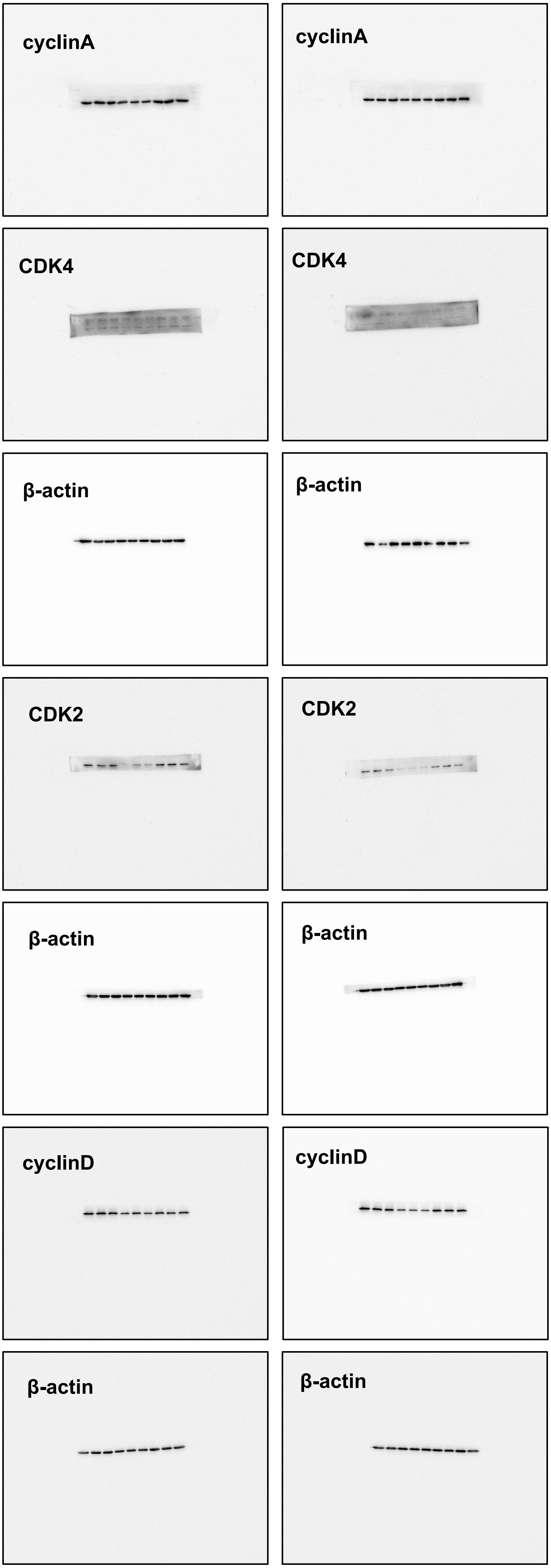
cyclinA
cyclinA
CDK4
CDK4
β-actin
β-actin
CDK2
CDK2
β-actin
β-actin
cyclinD
cyclinD
β-actin
β-actin

Figure4 A:

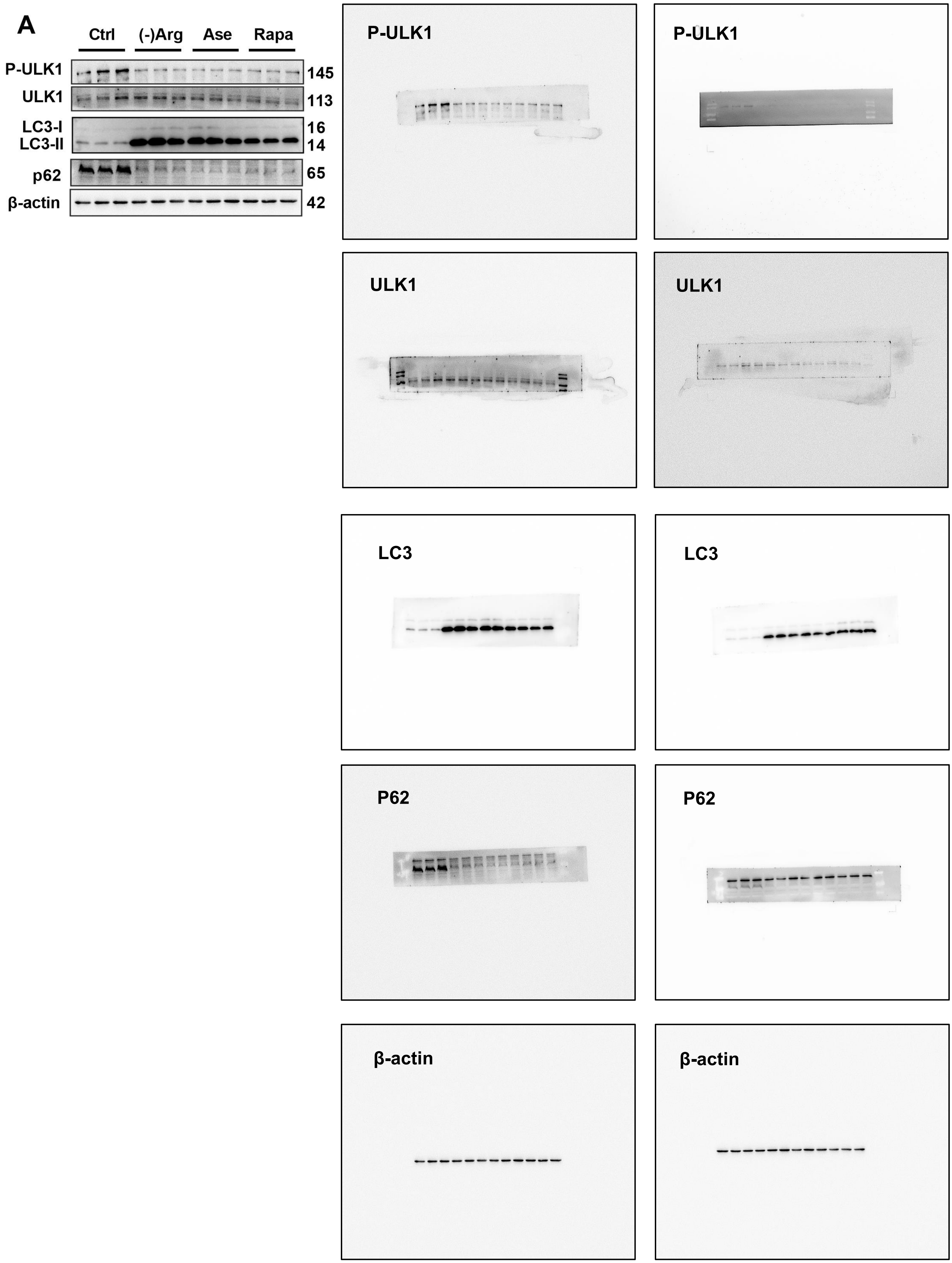
A
Ctrl
(-)Arg
Ase
Rapa
P-ULK1
145
ULK1
113
LC3-I
16
LC3-II
14
p62
65
β-actin
42
P-ULK1
P-ULK1
ULK1
ULK1
LC3
LC3
P62
P62
β-actin
β-actin

Figure4 B:

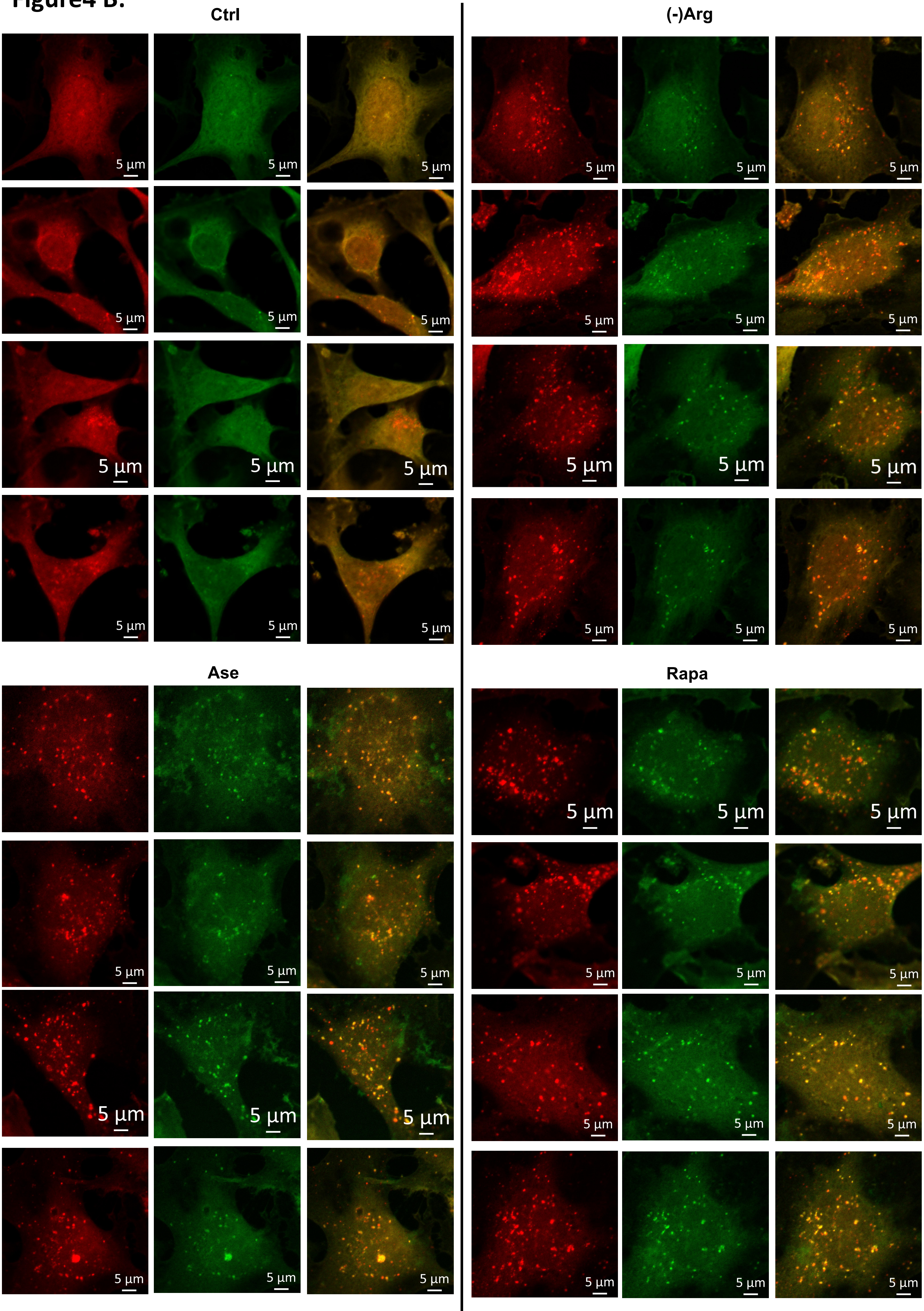

Figure4 C:

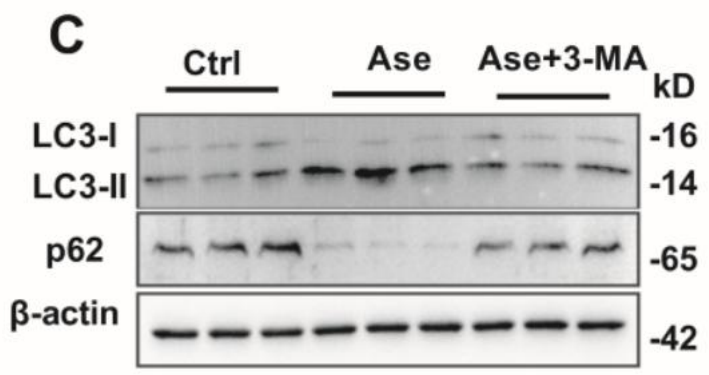
C
Ctrl
Ase
Ase+3-MA
kD
LC3-I
LC3-II
p62
β-actin
-16
-14
-65
-42

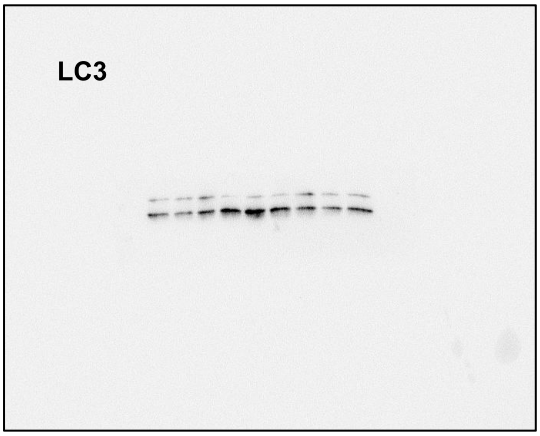
LC3

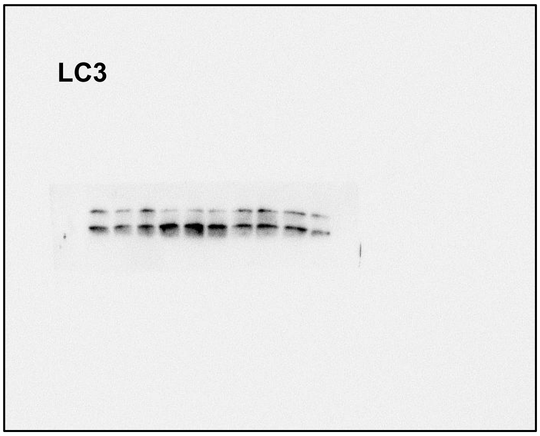
LC3

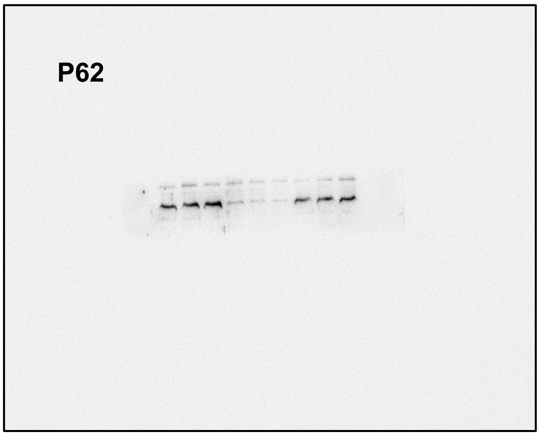
P62

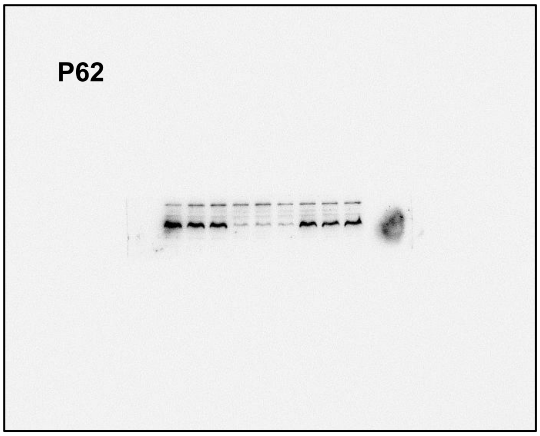
P62

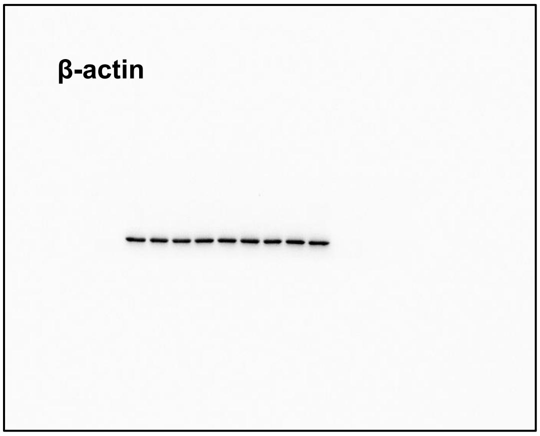
β-actin

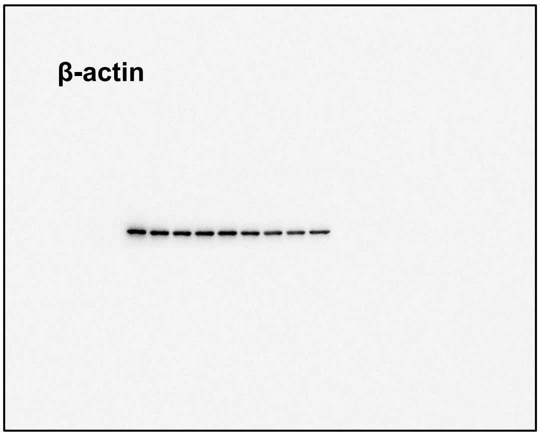
β-actin

Figure5 A:

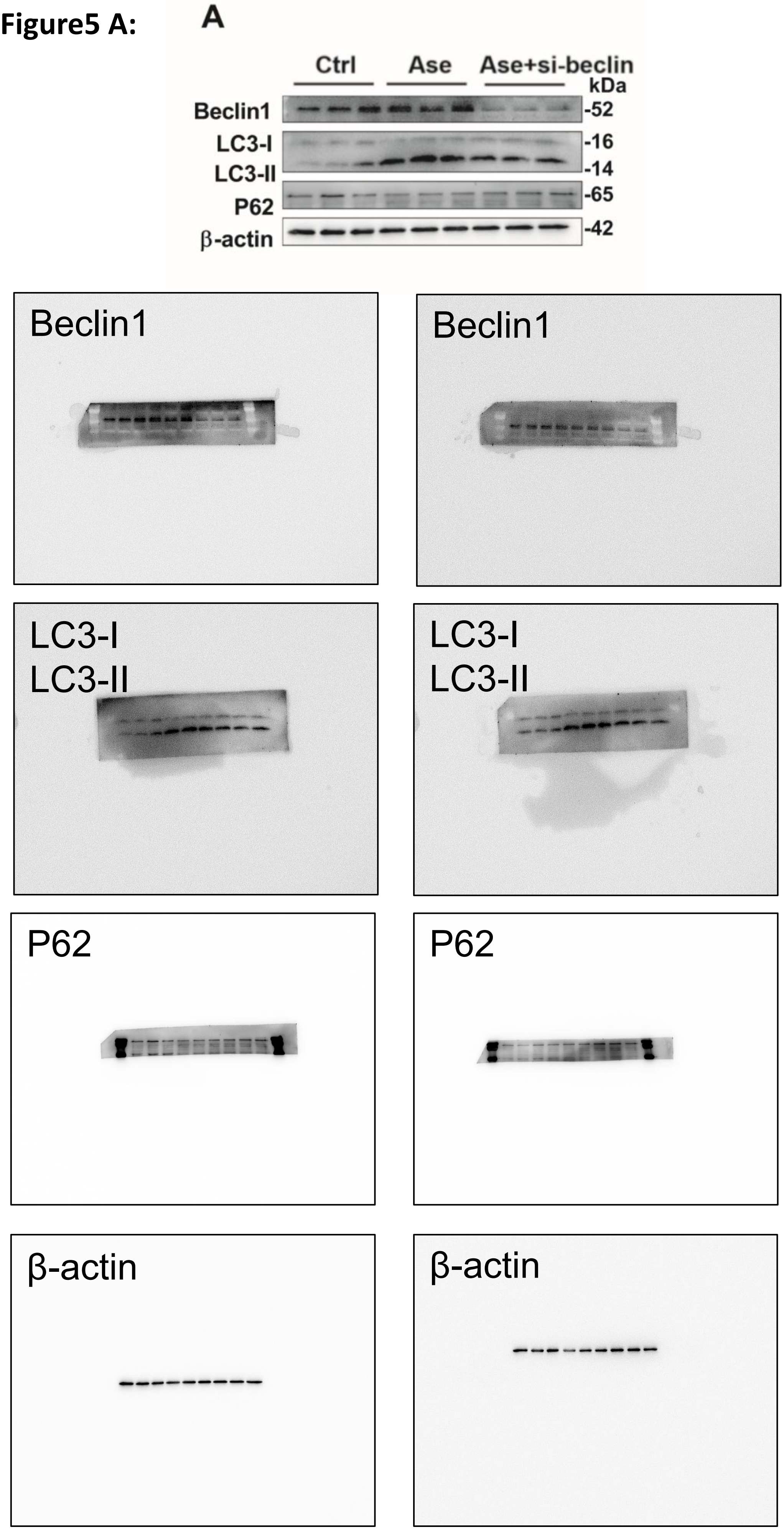

A
Ctrl
Ase
Ase+si-beclin
kDa
Beclin1
LC3-I
LC3-II
P62
β-actin
-52
-16
-14
-65
-42
Beclin1
Beclin1
LC3-I
LC3-II
LC3-I
LC3-II
P62
P62
β-actin
β-actin

Figure5 D:

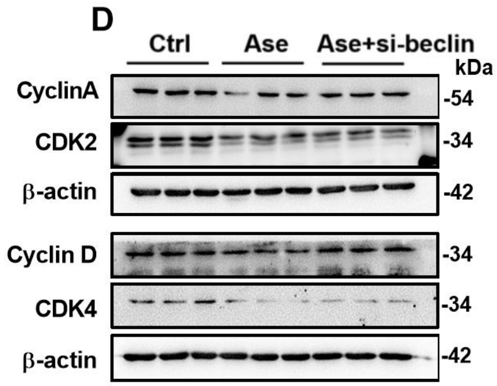
D
Ctrl
Ase
Ase+si-beclin
kDa
CyclinA
-54
CDK2
-34
β-actin
-42
Cyclin D
-34
CDK4
-34
β-actin
-42

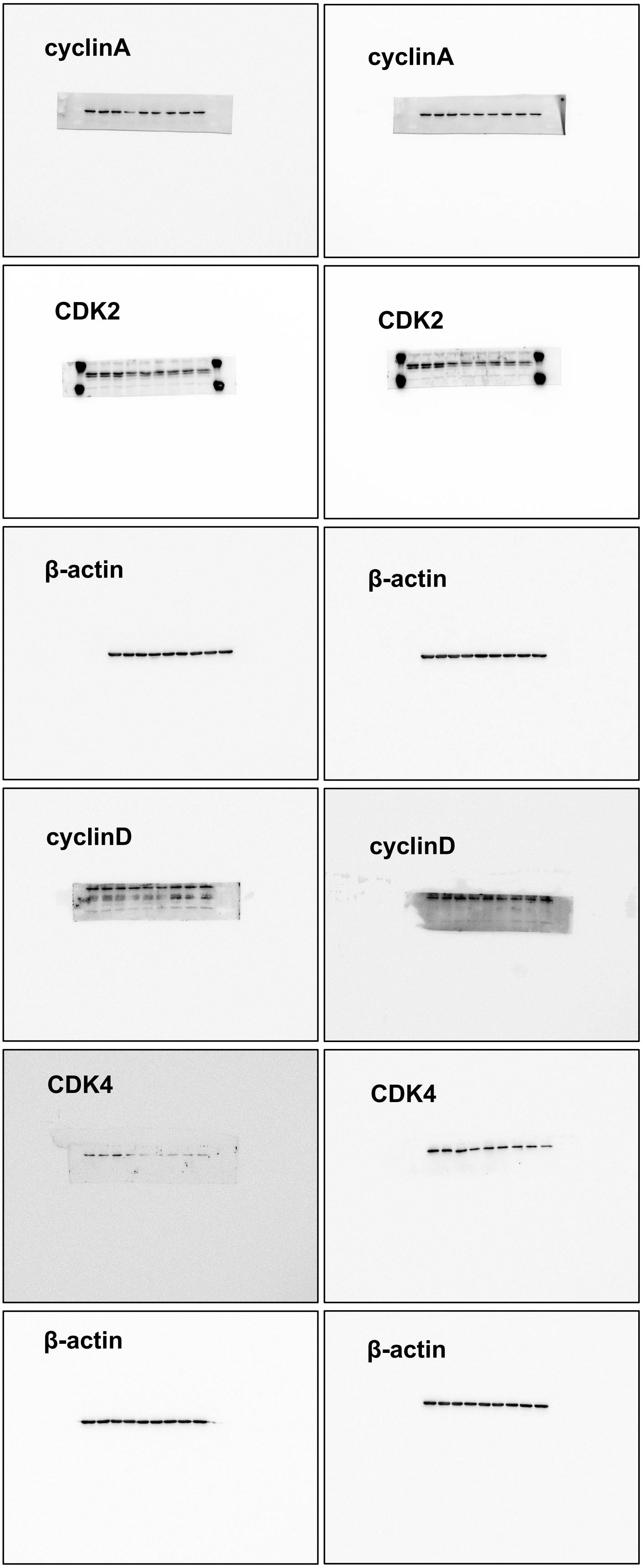
cyclinA
cyclinA
CDK2
CDK2
β-actin
β-actin
cyclinD
cyclinD
CDK4
CDK4
β-actin
β-actin

Figure6 D:

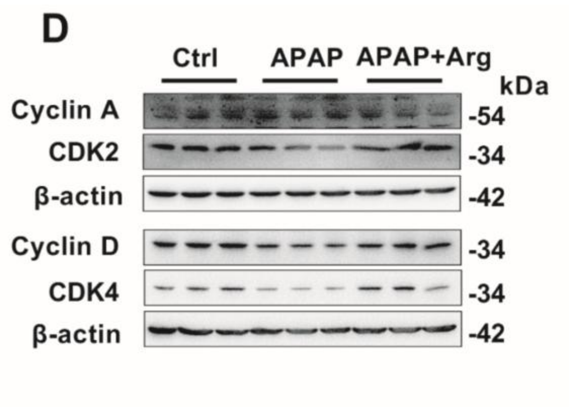
D
Ctrl
APAP
APAP+Arg
kDa
Cyclin A
-54
CDK2
-34
β-actin
-42
Cyclin D
-34
CDK4
-34
β-actin
-42

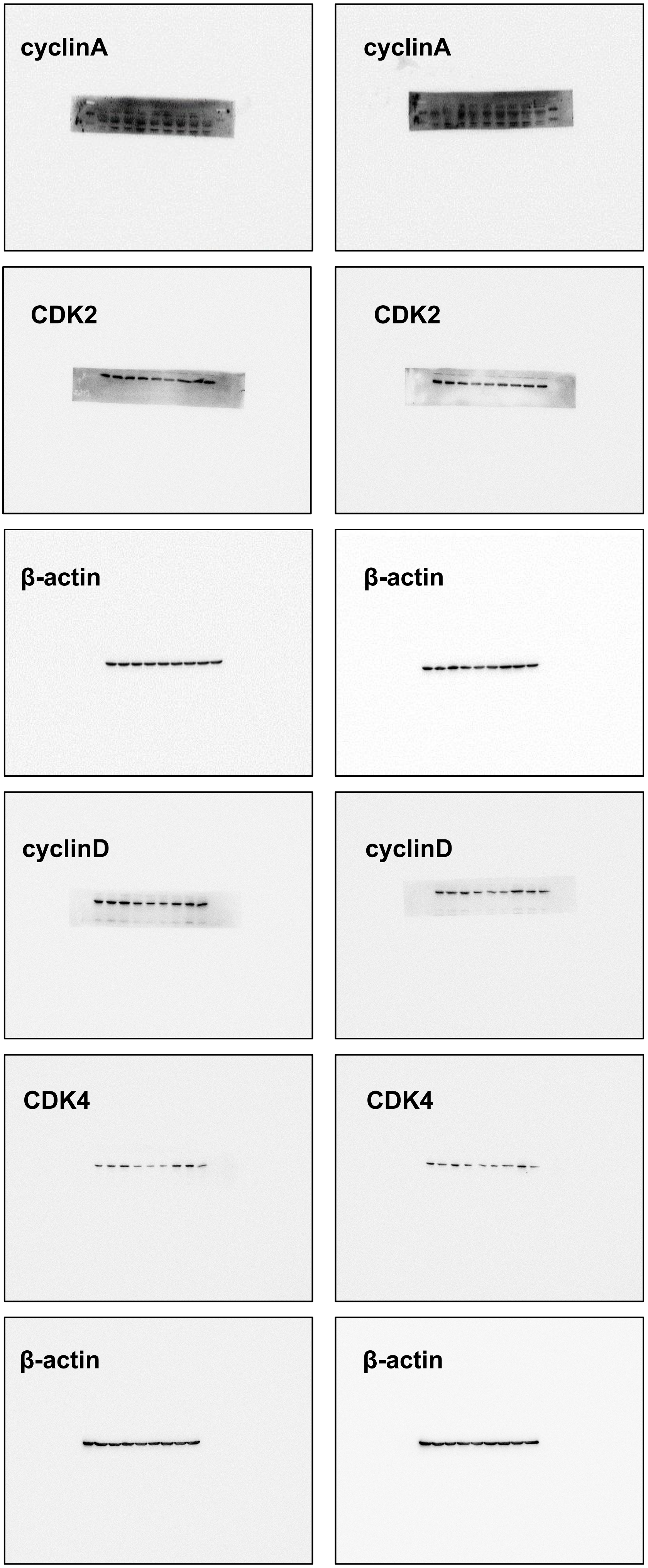
cyclinA
cyclinA
CDK2
CDK2
β-actin
β-actin
cyclinD
cyclinD
CDK4
CDK4
β-actin
β-actin

Figure6 H:

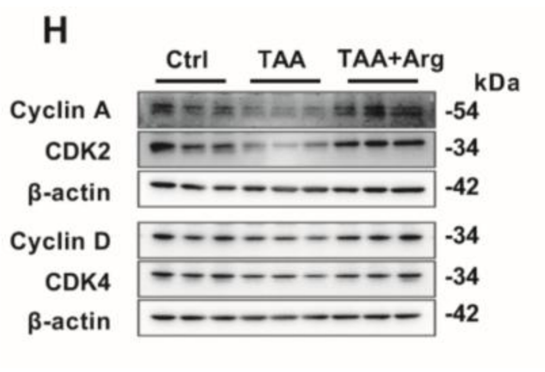
H
Ctrl
TAA
TAA+Arg
kDa
Cyclin A
-54
CDK2
-34
β-actin
-42
Cyclin D
-34
CDK4
-34
β-actin
-42

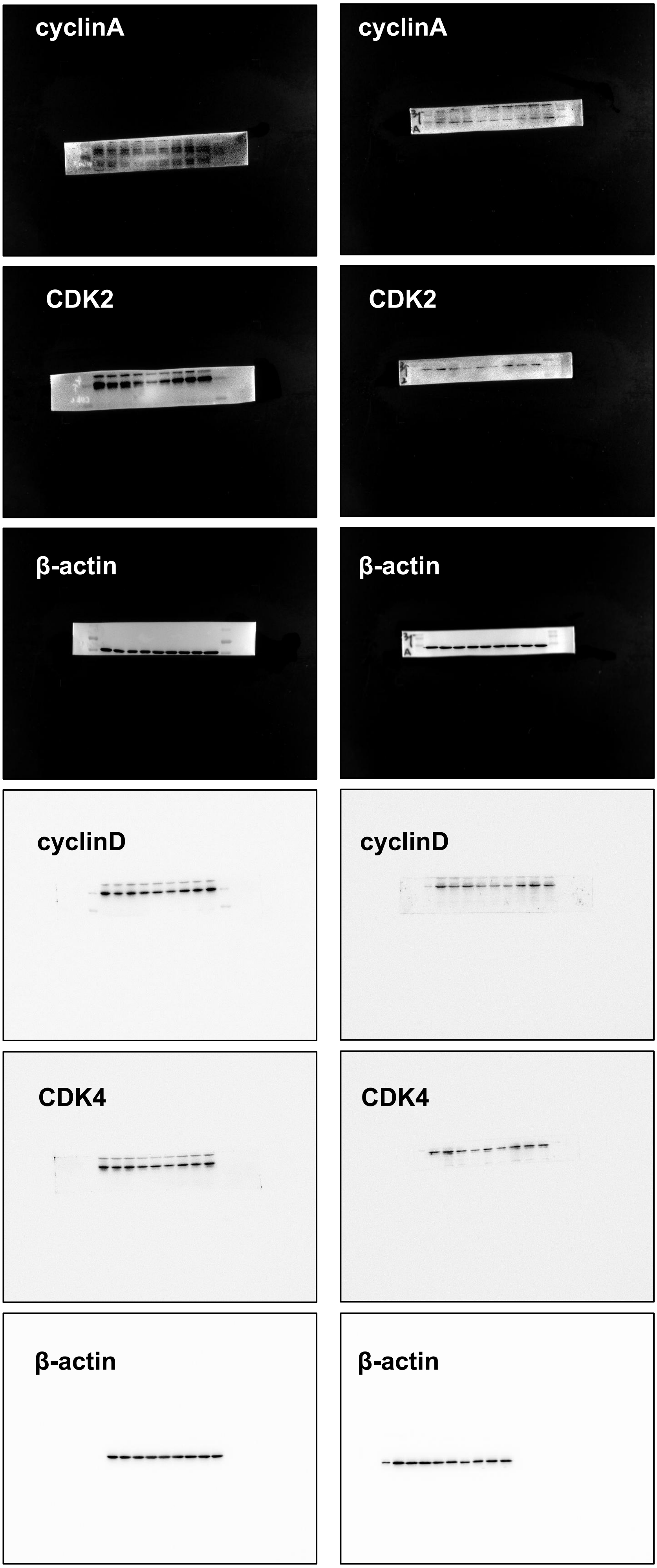
cyclinA
cyclinA
CDK2
CDK2
β-actin
β-actin
cyclinD
cyclinD
CDK4
CDK4
β-actin
β-actin

Figure7 D

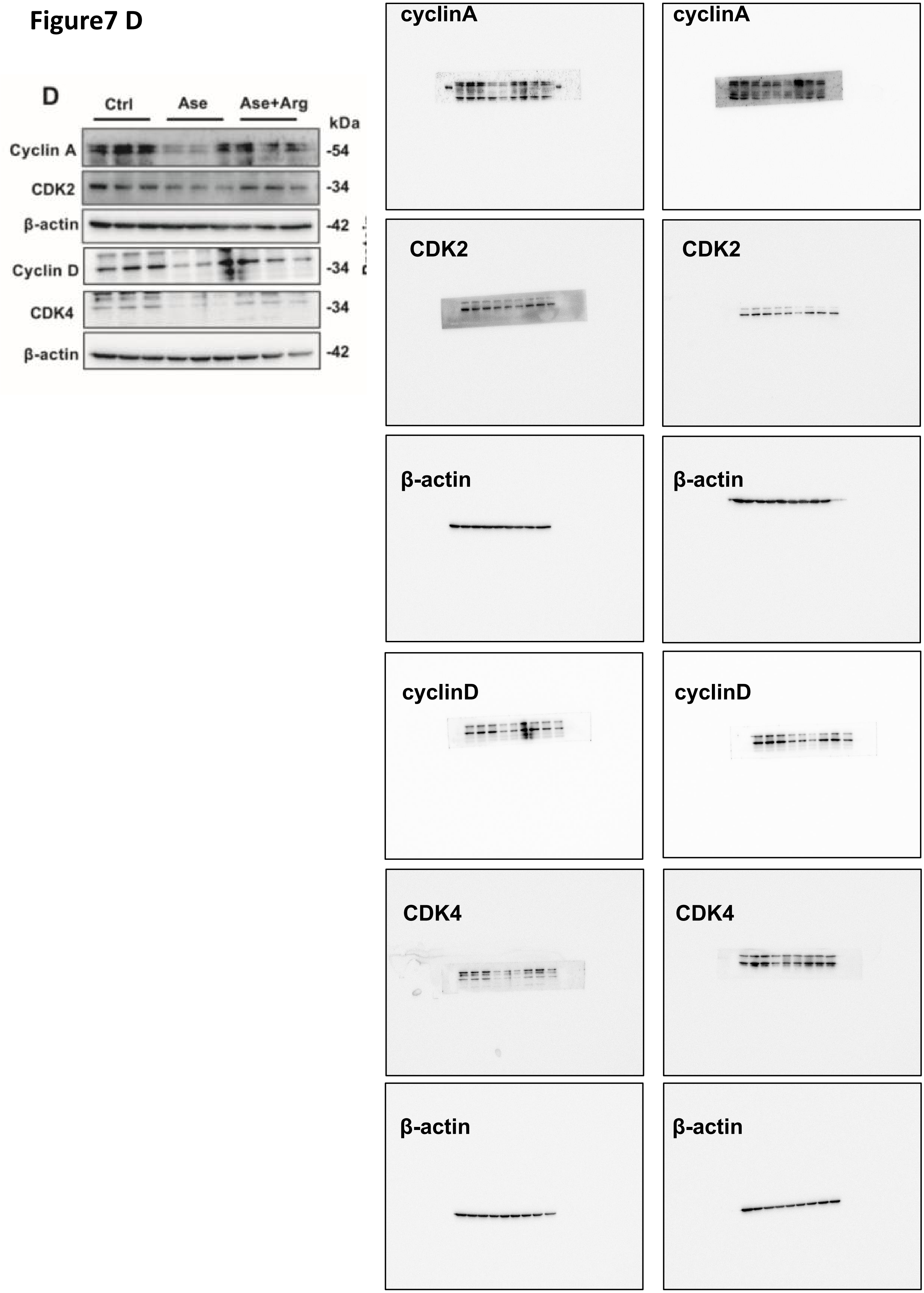
D
Ctrl
Ase
Ase+Arg
kDa
Cyclin A
-54
CDK2
-34
β-actin
-42
Cyclin D
-34
CDK4
-34
β-actin
-42
cyclinA
cyclinA
CDK2
CDK2
β-actin
β-actin
cyclinD
cyclinD
CDK4
CDK4
β-actin
β-actin

Figure7 E

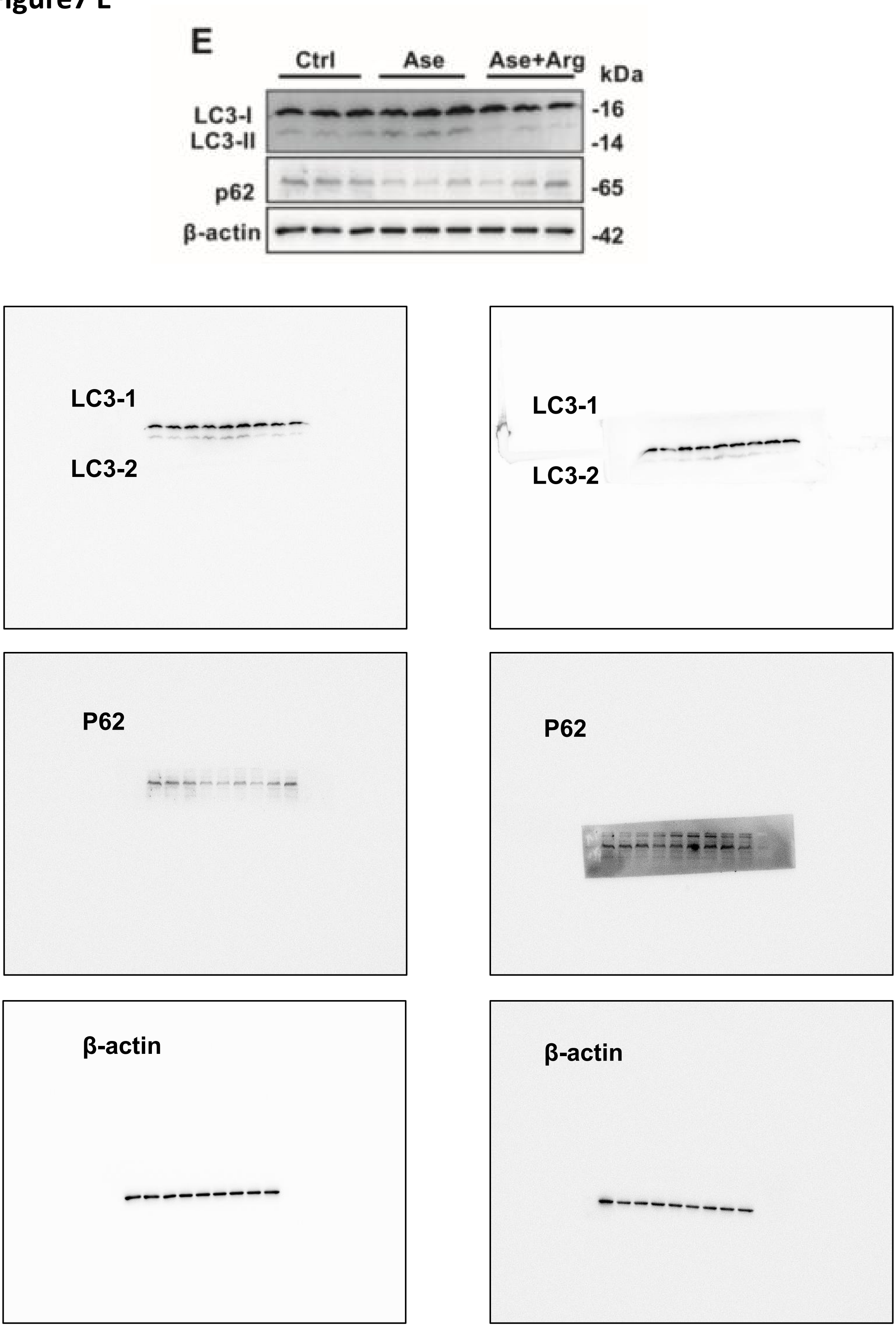

E
Ctrl
Ase
Ase+Arg
kDa
LC3-I
LC3-II
-16
-14
p62
-65
β-actin
-42
LC3-1
LC3-2
LC3-1
LC3-2
P62
P62
β-actin
β-actin

Figure7 F

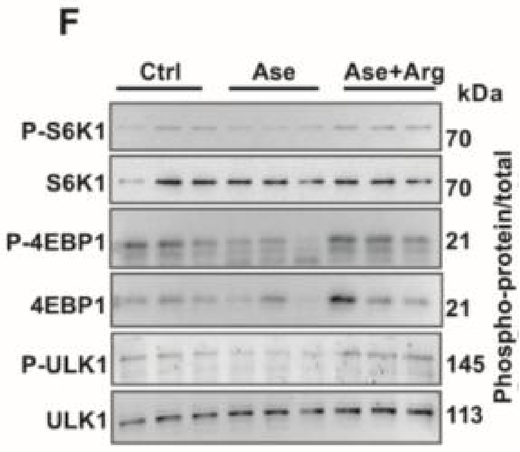
F
Ctrl
Ase
Ase+Arg
kDa
P-S6K1
70
S6K1
70
P-4EBP1
21
4EBP1
21
P-ULK1
145
ULK1
113
Phospho-protein/total

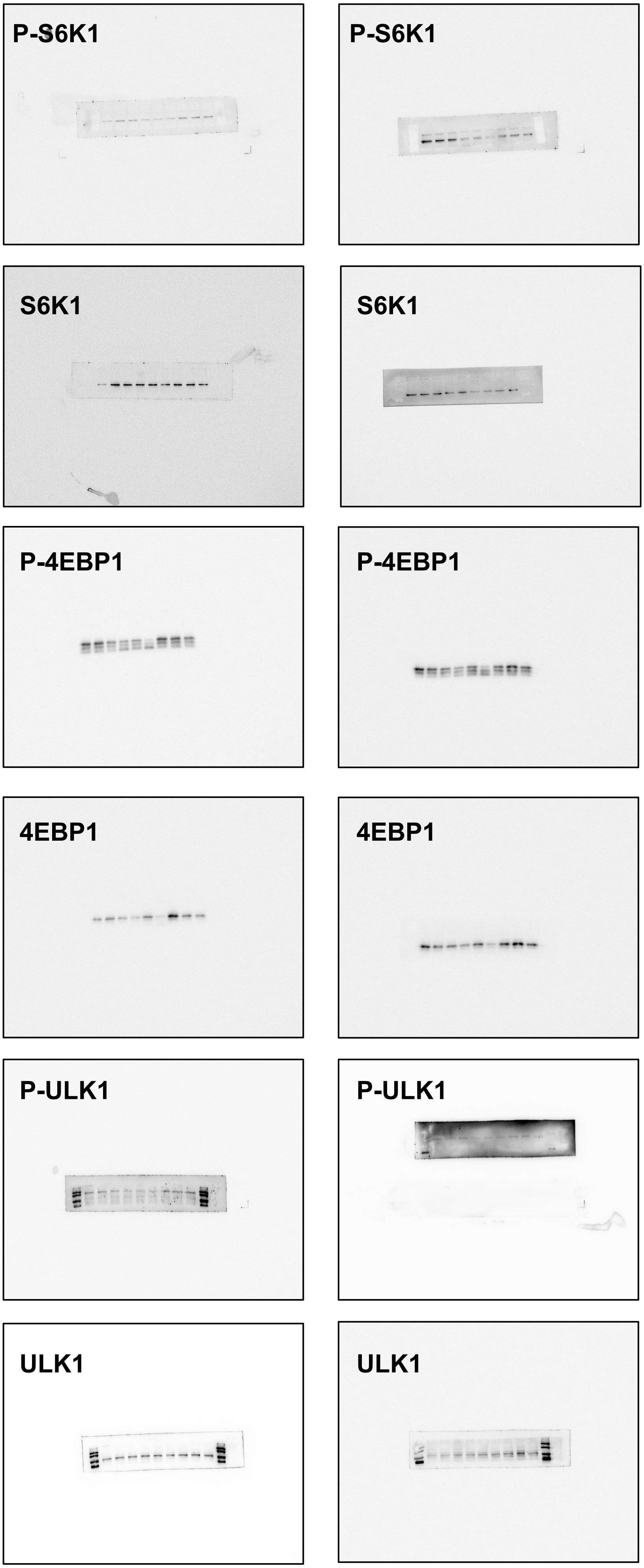
P-S6K1
P-S6K1
S6K1
S6K1
P-4EBP1
P-4EBP1
4EBP1
4EBP1
P-ULK1
P-ULK1
ULK1
ULK1
