# Supplementary material for: Acute liver failure-induced arginine deficiency impairs blood-brain barrier via inhibiting mTORC1-S6K1/4EBP1 pathway and inducing autophagy
Source: Cell Death Dis. 2025 Nov 17;16(1):842. doi: 10.1038/s41419-025-08152-4 (PMC12623816; doi:10.1038/s41419-025-08152-4)
Supplement: Supplementary file 2 — Extended Data Figures and Tables [file 41419_2025_8152_MOESM2_ESM.docx]

**Supplementary material for**

**Acute liver failure-induced arginine deficiency impairs blood-brain barrier via inhibiting mTORC1-S6K1/4EBP1 pathway and inducing autophagy**

Table of contents

Fig. S1....................................................................................................2

Fig. S2....................................................................................................3

Fig. S3....................................................................................................4

Fig. S4....................................................................................................5

Fig. S5....................................................................................................6

Fig. S6....................................................................................................7

Fig. S7....................................................................................................8

Fig. S8....................................................................................................9

Table. S1.................................................................................................10

Table. S2.................................................................................................11

Table. S3.................................................................................................11

Table. S4.................................................................................................12


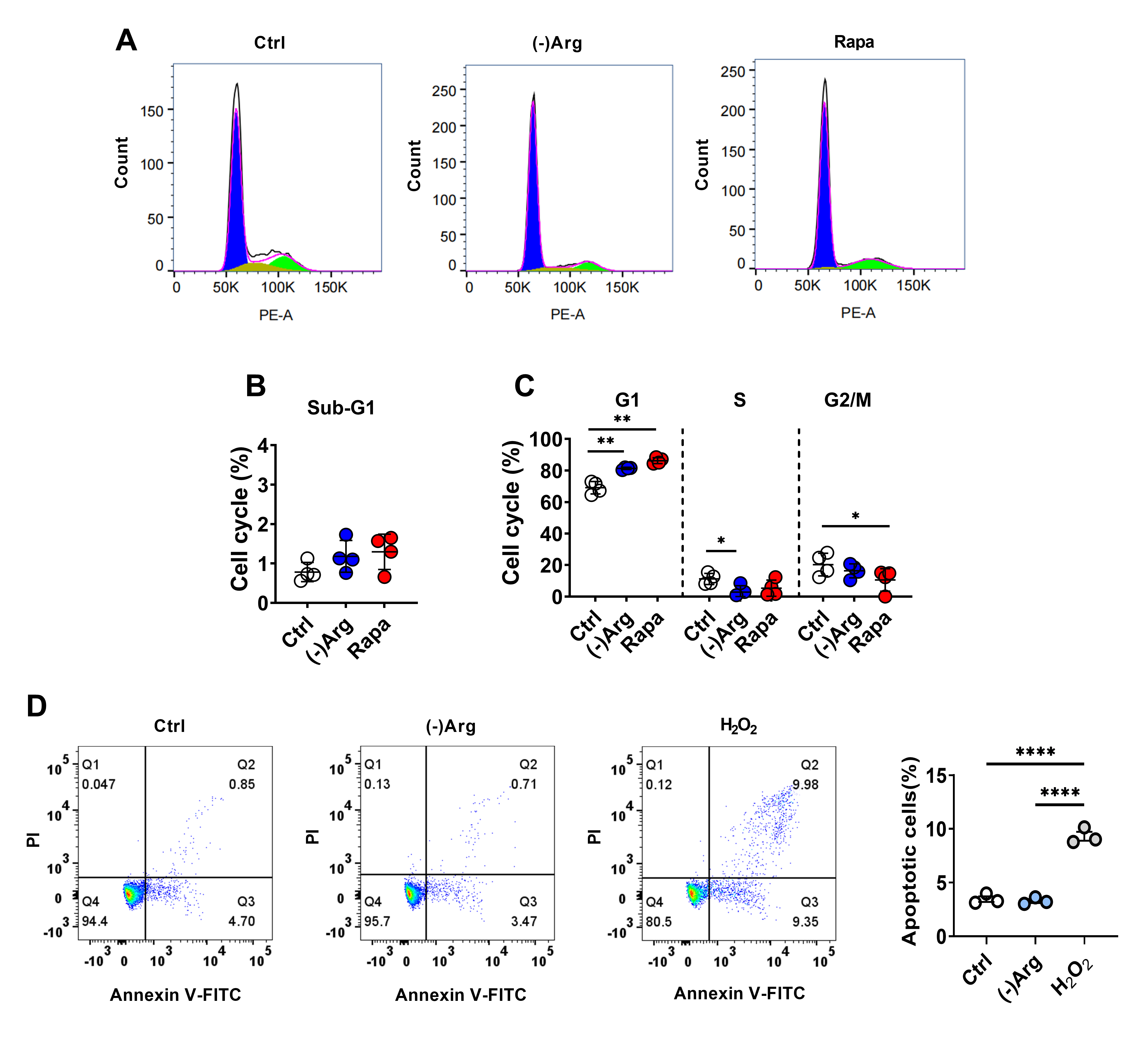


**Fig. S1.** **Arginine deficiency-mediated cell cycle arrest was independent of apoptosis.** (A-C) Cell cycle analysis on flow cytometer by PI staining of hCMEC/D3 cells treated with arginine-free culture ((-)Arg) or rapamycin (Rapa) (n=4). (D) Cell apoptosis analysis by Annexin V-FITC/PI staining of rBMECs cells treated with (-)Arg or H_2_O_2_ (n=3). Data are expressed as mean ± SD. Statistical significance was determined with the 1-way ANOVA followed by the Dunnett post hoc test. *p<0.05; **p<0.01.


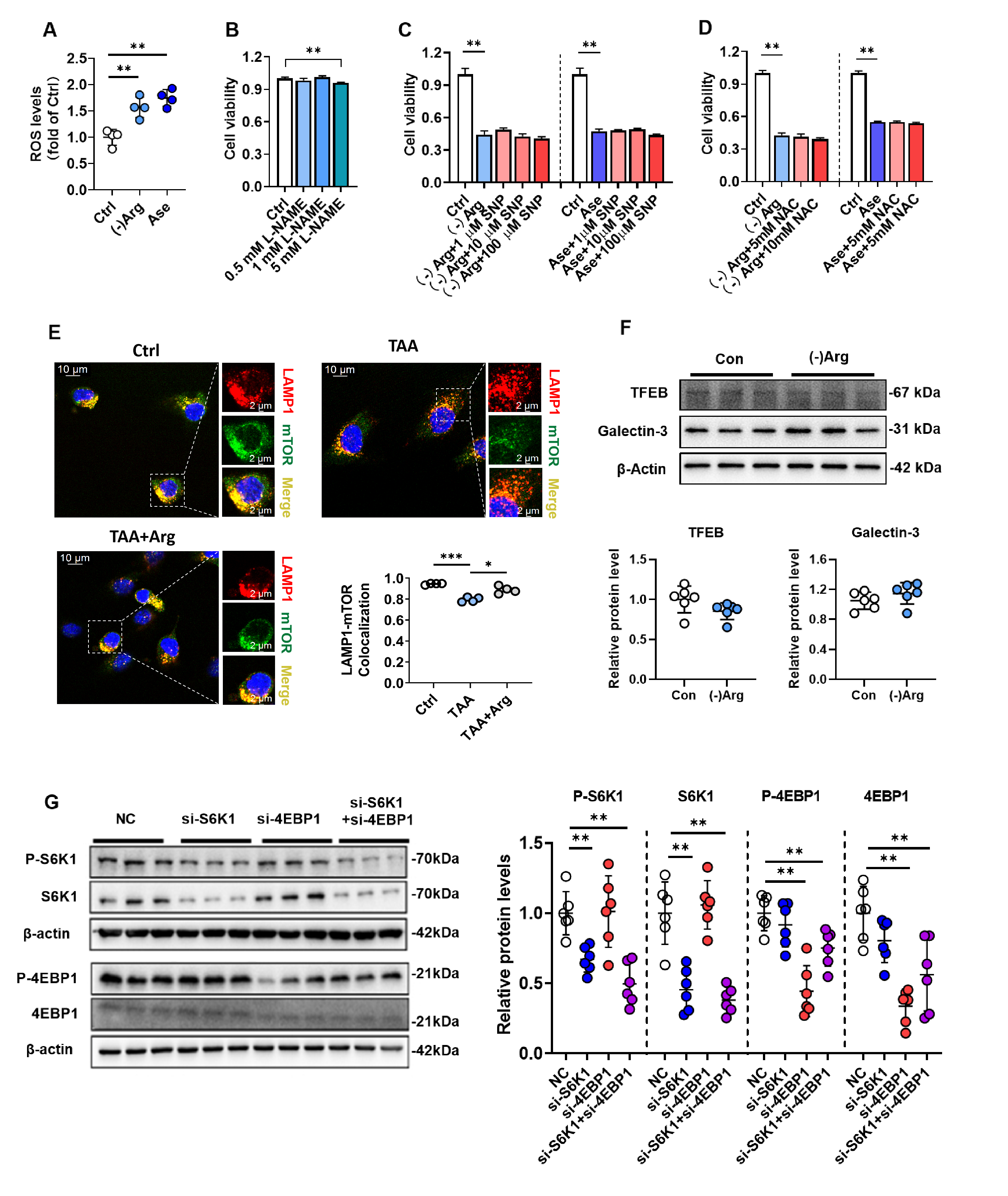


**Fig. S2.** **Involvement of NO-ROS and mTORC1 pathway in arginine deficiency-mediated cell damage in hCMEC/D3 cells.** (A) Relative ROS levels in hCMEC/D3 cells treated with Arg-free medium ((-)Arg) or 20 μg/mL arginase (Ase). (B) Effect of NO synthase inhibitor L-NAME on hCMEC/D3 cell viability. Effect of nitric oxide donor Sodium Nitroprusside (SNP) (C) and free radical scavenger N-acetylcysteine (NAC) (D) on hCMEC/D3 cell damage by (-)Arg or Ase. (E) Co-immunofluorescence of mTOR (green) and LAMP1 (red) in hCMEC/D3 cells treated by 50 μg/mL Arg and 10% Ctrl or TAA rat serum. (F) The effect of Arg deficiency on protein levels of TFEB and Galectin-3 in hCMEC/D3 cells. (G) Western blot of phosphorylated and total S6K1 and 4EBP1 in hCMEC/D3 cells silencing S6K1 and 4EBP1 (n=6). Data are expressed as mean ± SD. Statistical significance was determined with the 1-way ANOVA or t-test. *p<0.05; **p<0.01, n=4 for A-E, n=6 for F and G.


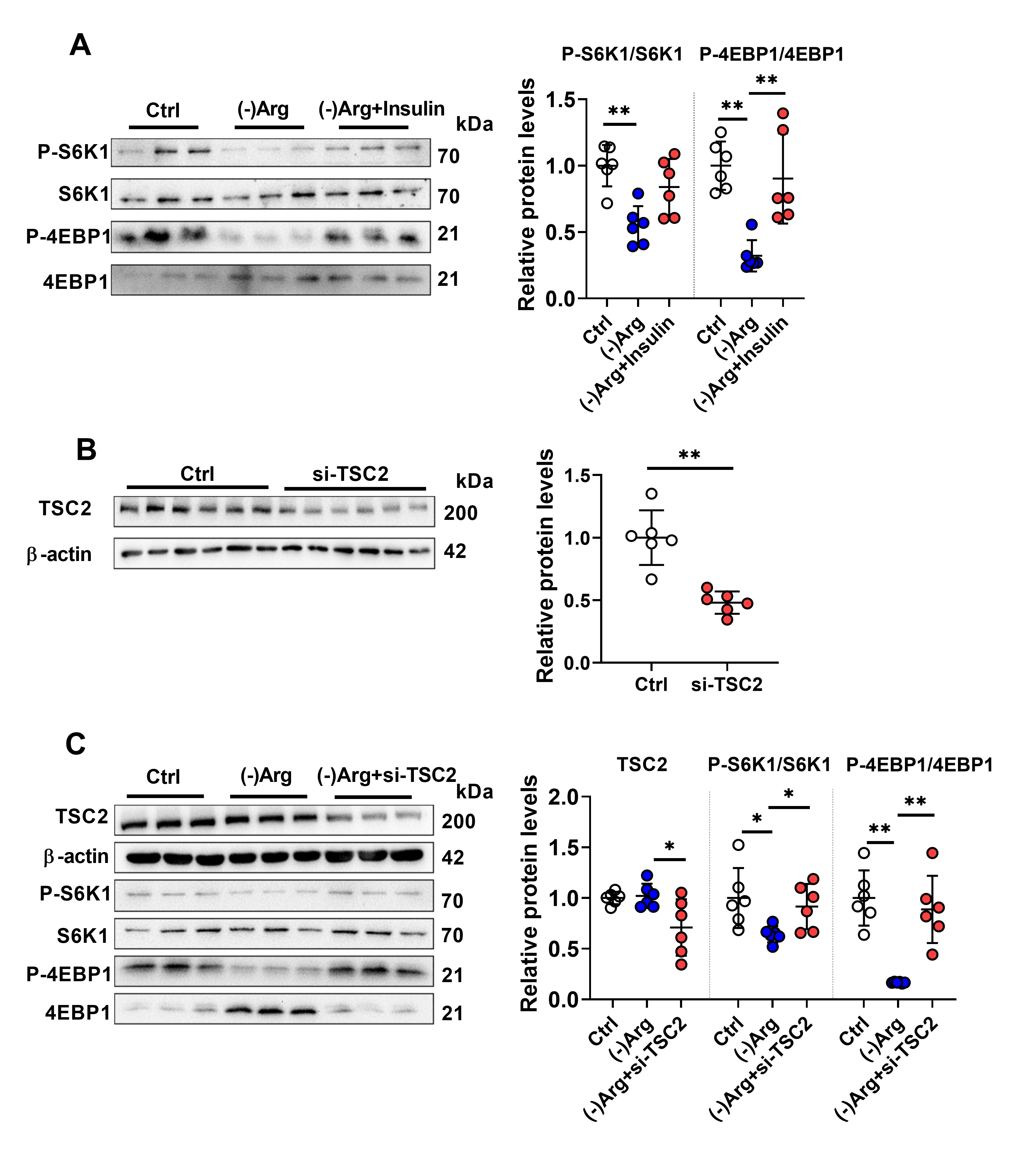


**Fig. S3.** **Effects of insulin and silencing TSC2 on phosphorylation of the S6K1 and 4EBP1.** (A) Phosphorylation of S6K1 and 4EBP1 in hCMEC/D3 cells treated with arginine-free medium ((-)Arg) and insulin. Effects of silencing TSC2 on TSC2 protein levels (B) and Arg deficiency-mediated reductions in P-S6K1/S6K1 and P-4EBP1/4EBP1 in hCMEC/D3 cells (C). Data are expressed as mean ± SD. Statistical significance was determined with the 1-way ANOVA or t-test. *p<0.05; **p<0.01, n=6.


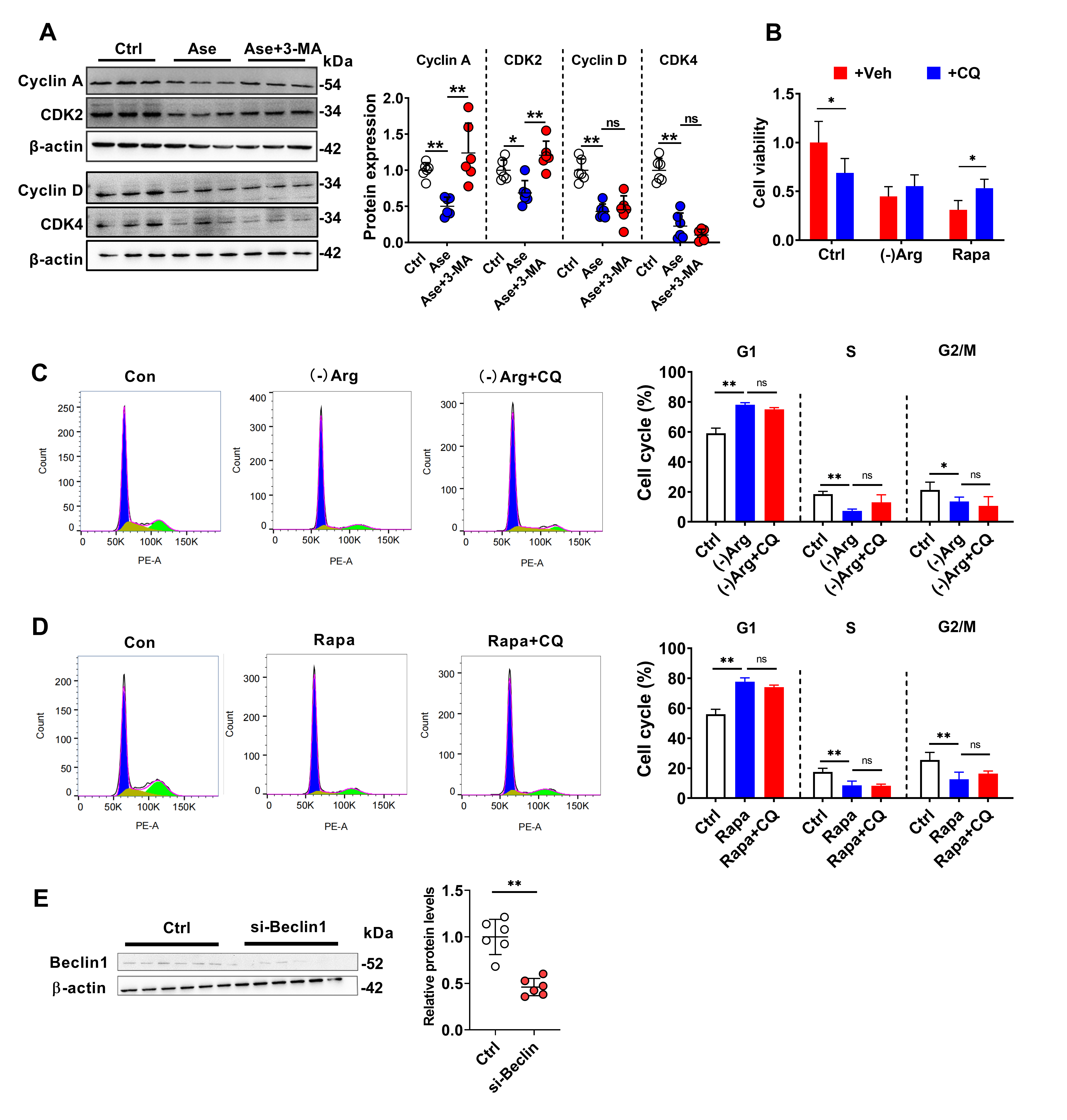


**Fig. S4. Effects of autophagy inhibitor on Arginine deficiency-mediated hCMEC/D3 cell cycle changes.** (A) Effects of autophagy inhibitor 3-methyladenine (3-MA) on cell cycle-related proteins expression in arginase (Ase)-treated hCMEC/D3 cells (n=6). Effects of autophagy inhibitor chloroquine (CQ) on hCMEC/D3 cell damage (B) and cell cycle arrest (C and D) caused by arginine-free medium ((-)Arg) or rapamycin (Rapa) (n=4). (E) Effects of silencing Beclin1 on Beclin1 protein levels (n=6). Data are expressed as mean ± SD. Statistical significance was determined with the 1-way ANOVA or t-test. *p<0.05; **p<0.01.


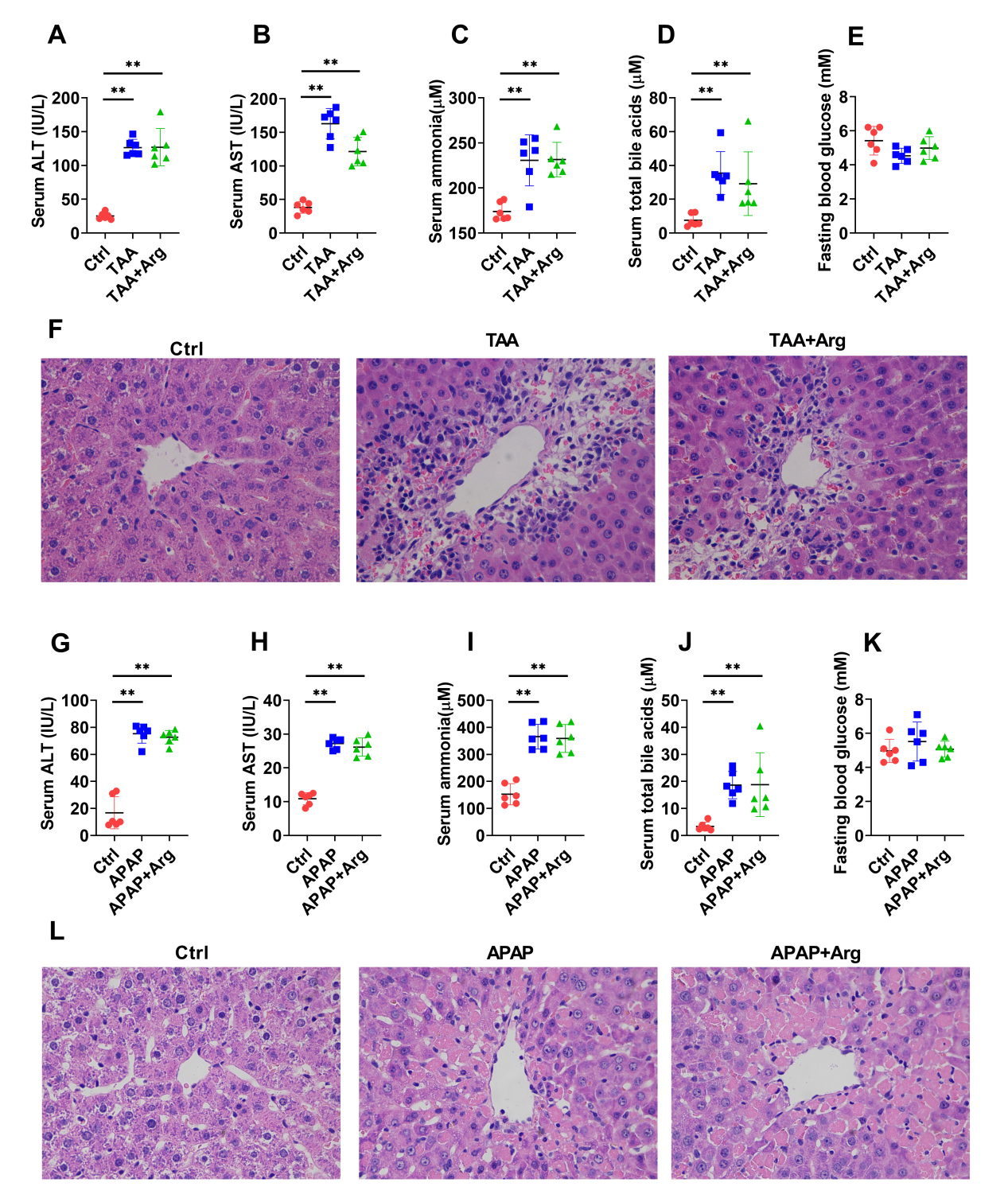


**Fig. S5. Determine of liver function in TAA or APAP-induced acute liver failure rats.** Serum ALT (A), AST (B), ammonia (C), total bile acids (D), and fasting blood glucose levels (E) in control rats (Ctrl), rats intraperitoneally receiving 3 doses of 300 mg/kg thioacetamide (TAA) and TAA rats orally supplemented with 0.18 g/kg Arg (TAA+Arg). (F) Histological features of TAA rats liver sections stained with H&E. (G-L) Serum ALT, AST, ammonia, total bile acids, and fasting blood glucose levels, and liver sections H&E stained results in control Ctrl rats, rats intraperitoneally receiving a single dose of 500 mg/kg acetaminophen (APAP) and APAP rats orally supplemented with 0.18 g/kg Arg (APAP+Arg). Data are expressed as mean±SD. Statistical significance was determined with the 1-way ANOVA. *p<0.05; **p<0.01, n=6.


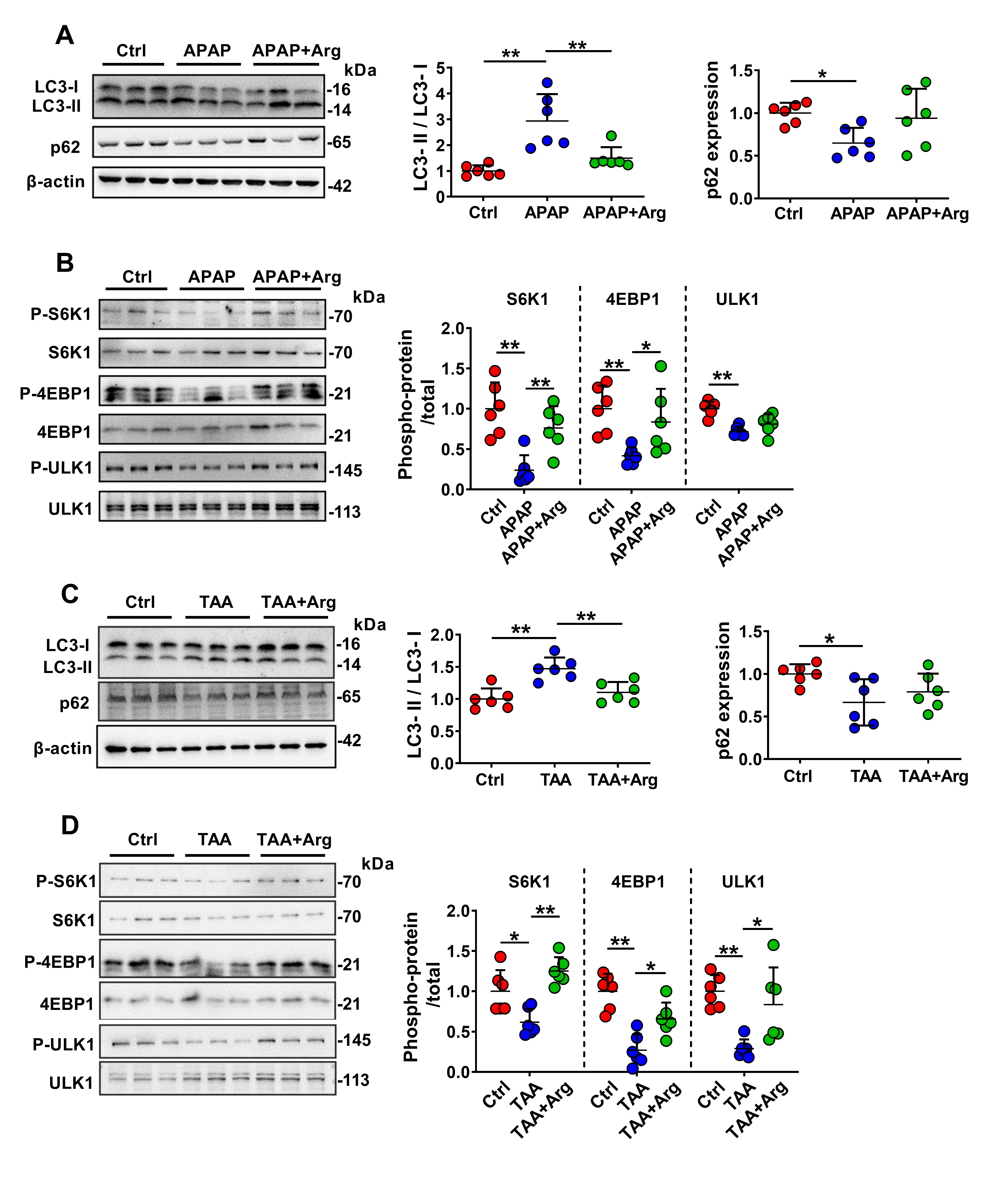


**Fig. S6. Arginine supplementation attenuated ALF-induced mTORC1-S6K1/4EBP1 pathway inhibition and autophagy induction.** Autophagy markers (LC3 and p62) (A), and mTORC1 pathway-related proteins (S6K1, 4EBP1, and ULK1) phosphorylation (A) in isolated cerebral microvessels of APAP and APAP+Arg rats. Autophagy markers (LC3 and p62) (C), and mTORC1 pathway-related proteins (S6K1, 4EBP1, and ULK1) phosphorylation (D) in isolated cerebral microvessels of TAA and TAA+Arg rats. Data are expressed as mean±SD. Statistical significance was determined with the 1-way ANOVA. *p<0.05; **p<0.01, n=6.

**
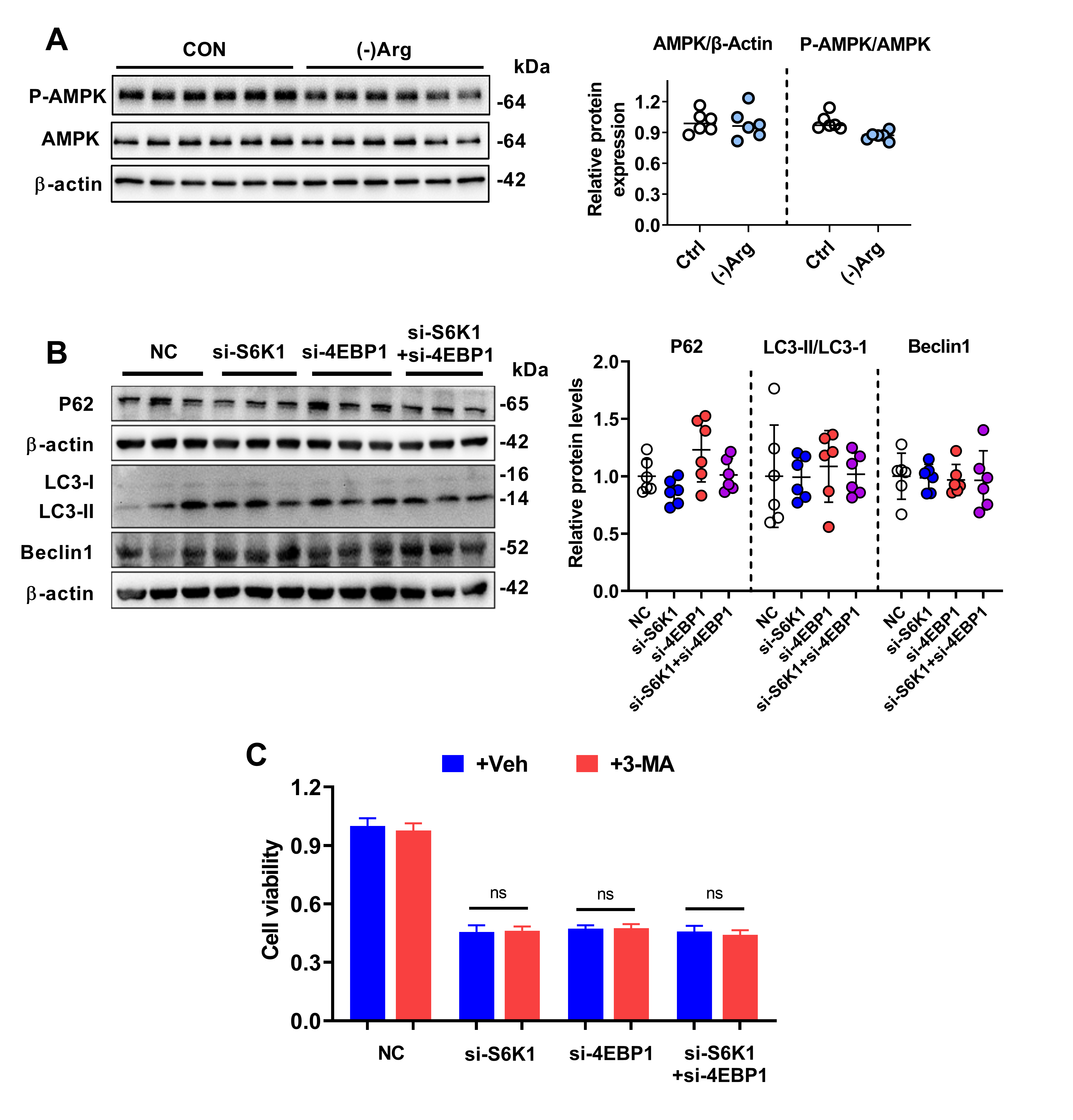
**

**Fig. S7.** (A) Arg deficiency unaffected AMPK expression or phosphorylation in hCMEC/D3 cells. Silencing S6K1 and 4EBP1 impaired hCMEC/D3 cells independent with autophagy induction: (B) Western blot of LC3, p62, and Beclin1 proteins in hCMEC/D3 cells silencing S6K1, 4EBP1, or S6K1 plus 4EBP1 (n=6). (C) Autophagy inhibitor 3-methyladenine (3-MA) did not attenuate BBB impairment by silencing S6K1 or 4EBP1 (n=4). Data are expressed as mean±SD. Statistical significance was determined with the 1-way ANOVA or t-test. *p<0.05; **p<0.01.


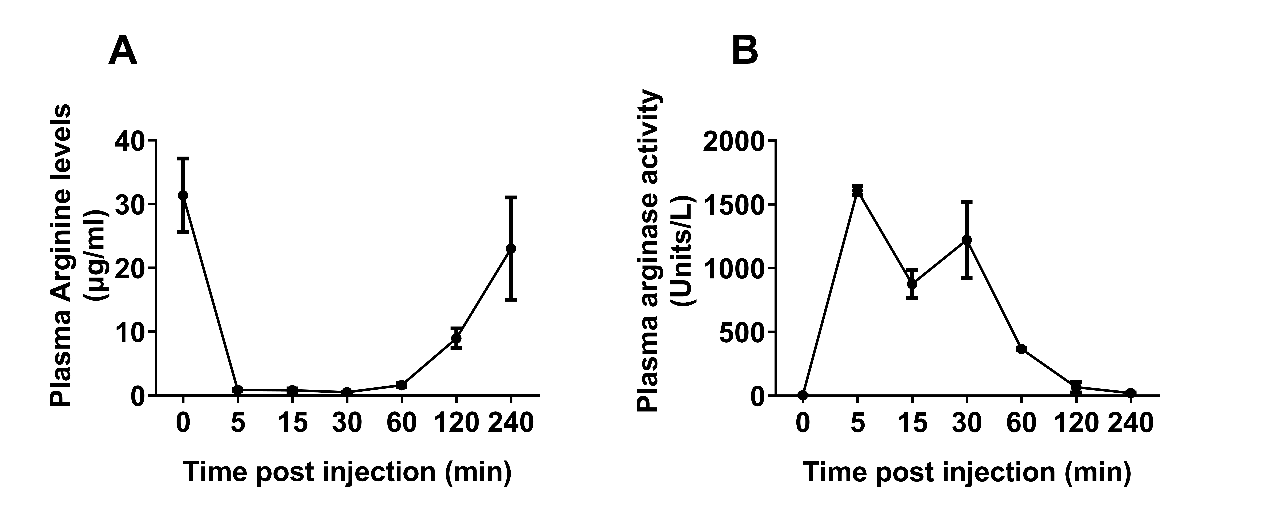


**Fig. S8. Plasma arginine levels (A) and arginase activity (B) changes in rats after mono-dose injection of arginase (71.25 units/kg) intravenously**. Data are expressed as mean±SD, n=3.

**Table S1: Antibodies**

| **Name** | **Supplier** | **Cat no.** | **Clone no.** |
| --- | --- | --- | --- |
| Cyclin A | SANTA CRUZ | sc-239 | BF683 |
| Cyclin D | SANTA CRUZ | sc-8396 | A-12 |
| CDK2 | SANTA CRUZ | sc-6248 | D-12 |
| CDK4 | SANTA CRUZ | sc-23896 | DCS-35 |
| Arginase Ⅰ | SANTA CRUZ | sc-47715 | 8C9 |
| S6K1 | SANTA CRUZ | sc-8418 | H-9 |
| Phospho-4E-BP1(Thr37/46) | SANTA CRUZ | sc-548713 | 62.Ser 65 |
| 4EBP1 | SANTA CRUZ | sc-9977 | P-1 |
| ULK1 | SANTA CRUZ | sc-390904 | F-4 |
| MAP LC3β | SANTA CRUZ | sc-271625 | G-2 |
| P62(SQSTM1) | SANTA CRUZ | sc-28359 | D-3 |
| CD31 (PECAM-1) | SANTA CRUZ | sc-376764 | H-3 |
| BECN1/Beclin-1 | SANTA CRUZ | sc-48341 | E-8 |
| Phospho-p70 S6K(Thr389) | Cell Signaling Technology | 9234 | 108D2 |
| Phospho-4E-BP1(Thr37/46) | Cell Signaling Technology | 2855 | 236B4 |
| Phospho-ULK1(Ser757& | Cell Signaling Technology | 14202 | D7O6U |
| Ki-67 | Cell Signaling Technology | 9129 | D3B5 |
| LAMP1 | Cell Signaling Technology | 15665 | D4O1S |
| Tuberin/TSC2 | Cell Signaling Technology | 4308 | D93F12 |
| β-actin | Proteintech | 66009-1-Ig | 2D4H5 |
| Anti-mouse IgG (HRP-linked) | Cell Signaling Technology | 7076 |  |
| Anti-rabbit IgG (HRP-linked) | Cell Signaling Technology | 7074 |  |
| Goat anti-rabbit IgG (Alexa Flour 488 conjugated) | Abcam | Ab150077 |  |
| Goat anti-mouse IgG (Alexa Flour 594 conjugated) | Invitrogen | A-21125 |  |

**Table S2: Sequence based reagents**

| **Name** | **Sequence** | **Supplier** |
| --- | --- | --- |
| Human S6K1 siRNA | 5’-GGCAAUGAUAGUAAGAAAUTT | XINJIA MEDICAL |
| Human 4EBP1 siRNA | 5’-GCAAUAGCCCAGAAGAUAATT | XINJIA MEDICAL |
| Human TSC2 siRNA | 5’-AAAGUUCACCUACUGCUGGCA | GenePharma |
| Negative control siRNA | 5’-UUCUCCGAACGUGUCACGUTT | XINJIA MEDICAL |
| Human BECLIN-1 siRNA | 5′-GTGCTCCTGTGGAATGGAAT | GenePharma |

**Table S3: Software**

| **Software name** | **Manufacturer** | **Version** |
| --- | --- | --- |
| Graphpad Prism | GraphPad Software | 6.01 |
| Image J | NIH | 1.51j8 |
| FlowJo | BD Life Science | 10.4 |
| Image-Pro Plus | MEDIA CYBERNETCS | 6.0 |
| ZEN | Carl Zeiss Microscopy GmbH | 1. (blue edition) |

**Table S4: Other (e.g. drugs, proteins, vectors etc.)**

| **Drug, proteins** |  |  |
| --- | --- | --- |
| L-Arginase from bovine liver | Sigma-Aldrich | 9000-96-8 |
| Fluorescein sodium (Flu) | Aladdin | F105615 |
| N-Acetyl-L-Cysteine (NAC) | Adamas-beta | 70601A |
| 3-Methyladenine (3-MA) | Adamas-beta | 61446A |
| N'-Nitro-L-arginine-methyl ester hydrochloride (L-NAME) | Adamas-beta | 61334A |
| Sodium nitroprusside (SNP) | Sinopharm Chemical Reagent | 20040462 |
| Phthaldialdehyde (OPA) | Adamas-beta | 73745B |
| 3-Mercaptopropionic acid | Adamas-beta | 13485A |
| 70kDa dextran | TCI | D1449 |
| L-Arginine | Adamas-beta | 80826A |
| N^ω^-Hydroxy-nor-L-arginine (nor-NOHA) | Sigma-Aldrich | 399275 |
| Lipofectamine™ 3000 | Invitrogen | L3000015 |
| Insulin from bovine pancreas | Aladdin | 11070-73-8 |
| Rapamycin(rapa) | Aladdin | 53123-88-9 |
| Thioacetamide(TAA) | macklin | 62-55-5 |
| Acetaminophen(APAP) | macklin | 103-90-2 |
|  |  |  |
| **Assay kits** |  |  |
| CCK-8 Kit | Beyotime Biotechnology | C0042 |
| BCA protein assay Kit | Beyotime Biotechnology | P0010S |
| YF®488 Click-iT EdU Assay Kit | US EVERBRIGHT INC | C6015 |
| Cell cycle Analysis Kit | YEASEN Biotech | 40301ES50 |
| Arginase activity Kit | Sigma-Aldrich | MAK112 |
| Reactive oxiygen species AssayKit | Nanjing Jiancheng Bioengineering Institute | E004-1-1 |
| Nitric oxide detection kit | Beyotime | S0021S |
|  |  |  |
| **Vectors** |  |  |
| mRFP-GFP-LC3 adenoviral vectors | HanBio Technology | HB-AP2100001 |
|  |  |  |
| **Others** |  |  |
| RPMI-1640 medium | Invitrogen | 31800022 |
| RPMI-1640 medium for SILAC | Invitrogen | 88365 |
| Fetal bovine serum (FBS) | Invitrogen | 12483020 |
